# Slow cooling and highly efficient extraction of hot carriers in colloidal perovskite nanocrystals

**DOI:** 10.1038/ncomms14350

**Published:** 2017-02-08

**Authors:** Mingjie Li, Saikat Bhaumik, Teck Wee Goh, Muduli Subas Kumar, Natalia Yantara, Michael Grätzel, Subodh Mhaisalkar, Nripan Mathews, Tze Chien Sum

**Affiliations:** 1 Department of Physics and Applied Phyics, School of Physical and Mathematical Sciences, Nanyang Technological University, 21 Nanyang Link, SPMS-PAP 03-05, Singapore 637371, Singapore; 2 Energy Research Institute @ NTU (ERI@N), Research Techno Plaza, X-Frontier Block, Level 5, Singapore 637553, Singapore; 3 Laboratory of Photonics and Interfaces, Department of Chemistry and Chemical Engineering, Swiss Federal Institute of Technology, Station 6, CH-1015 Lausanne, Switzerland; 4 School of Materials Science and Engineering, Nanyang Technological University, 50 Nanyang Avenue, Singapore 639798, Singapore

## Abstract

Hot-carrier solar cells can overcome the Shockley-Queisser limit by harvesting excess energy from hot carriers. Inorganic semiconductor nanocrystals are considered prime candidates. However, hot-carrier harvesting is compromised by competitive relaxation pathways (for example, intraband Auger process and defects) that overwhelm their phonon bottlenecks. Here we show colloidal halide perovskite nanocrystals transcend these limitations and exhibit around two orders slower hot-carrier cooling times and around four times larger hot-carrier temperatures than their bulk-film counterparts. Under low pump excitation, hot-carrier cooling mediated by a phonon bottleneck is surprisingly slower in smaller nanocrystals (contrasting with conventional nanocrystals). At high pump fluence, Auger heating dominates hot-carrier cooling, which is slower in larger nanocrystals (hitherto unobserved in conventional nanocrystals). Importantly, we demonstrate efficient room temperature hot-electrons extraction (up to ∼83%) by an energy-selective electron acceptor layer within 1 ps from surface-treated perovskite NCs thin films. These insights enable fresh approaches for extremely thin absorber and concentrator-type hot-carrier solar cells.

## Abstract (web summary)

Harvesting excess energy from above-band gap photons could lead to solar cells which exceed conventional efficiency limits. Li *et al*., study hot carrier cooling in hybrid perovskite materials with reduced dimensionality using transient absorption spectroscopy and demonstrate efficient hot-electron extraction in such systems.

Thermodynamic calculations reveal that single junction solar cell conversion efficiencies can reach around 66% under 1 sun illumination if the excess energy of hot photogenerated carriers is used before they cool to the lattice temperature^1^. The key for effective extraction of hot-carrier energies is to retard the hot-carrier cooling. It was commonly believed that energy and momentum conservation together with improbability of multi-phonon processes would cause the phonon bottleneck that slows down hot-carrier cooling in semiconductor nanoscale systems^2^,^3^,^4^. However, further investigations revealed that the cooling rate becomes faster as dimensionality decreases for nanoscale inorganic semiconductors. Alternative rapid relaxation pathways (for example, intraband Auger-type energy transfer^5^,^6^ (Supplementary Fig. 1a), atomic fluctuations and surface effects^7^,^8^) were found to be highly effective in negating this perceived phonon bottleneck at low carrier densities. The reduced dimensionality also brings about competing effects at higher carrier densities: interband Auger recombination^9^. This latter Auger re-excitation process (also known as Auger heating, Supplementary Fig. 1c) would decelerate the hot-carrier cooling processes^10^,^11^. Consequently, hot-carrier cooling in nanoscale inorganic semiconductors is convolved with a complex interplay of disparate mechanisms. To date, it remains extremely challenging to achieve slow hot-carrier cooling even with strongly quantum-confined inorganic semiconducting nanocrystals (NCs)^2^. Despite the advances in theory and materials synthesis, practical hot-carrier colloidal NC photovoltaics remain elusive. Although slow intraband hot-electron cooling was found in the lowest excited levels (1P_e_−1S_e_) in strongly confined PbSe quantum dots^12^, the confined hot electrons had to be separated from holes with exceptionally well-engineered epitaxial grown multi-shells, to reduce the abovementioned competing relaxation pathways. Nevertheless, the multi-shells would complicate subsequent charge extraction. It is therefore necessary to design NCs that can simultaneously fulfill both criteria of slow hot carrier cooling and efficient charge extraction.

Organic–inorganic lead halide perovskite semiconductors (for example, MAPbX_3_, MA=CH_3_NH_3_, X=I, Br or Cl) have recently emerged as a leading contender in low-cost high-performance solar cells^13^,^14^,^15^,^16^. Recent observations of a hot-phonon bottleneck effect^17^,^18^ in MAPbI_3_ thin films at high carrier densities suggest that lead halide perovskites are also promising candidates for developing hot-carrier solar cells. Emulating semiconductor nanoscience, an interesting question would be whether the hot-carrier cooling rate in halide perovskites could be further modulated through confinement effects. Here, the hot-carrier cooling dynamics and mechanisms in colloidal methylammonium lead bromide (MAPbBr_3_) NCs of different sizes (with mean radius around 2.5–5.6 nm) (see transmission electron microscopy (TEM) images in Supplementary Fig. 2) and their bulk-film counterpart (see scanning electron microscopy (SEM) image in Supplementary Fig. 3) were compared using room-temperature transient absorption (TA) spectroscopy. Our results revealed that the weakly confined MAPbBr_3_ NCs (Supplementary Fig. 4 and Supplementary Note 1) are very promising hot-carrier absorber materials as they possess much higher hot-carrier temperatures and longer cooling times (as compared with typical perovskite bulk films under comparable photoexcitation conditions). This is attributed to their intrinsic phonon bottleneck and Auger-heating effects at low and high carrier densities, respectively. Importantly, the hot carriers can be efficiently extracted from MAPbBr_3_ NC thin films at room temperature by using a molecular semiconductor as an energy selective contact.

## Results

### Extraction of hot-carrier temperatures from TA spectra

**Figure 1:**
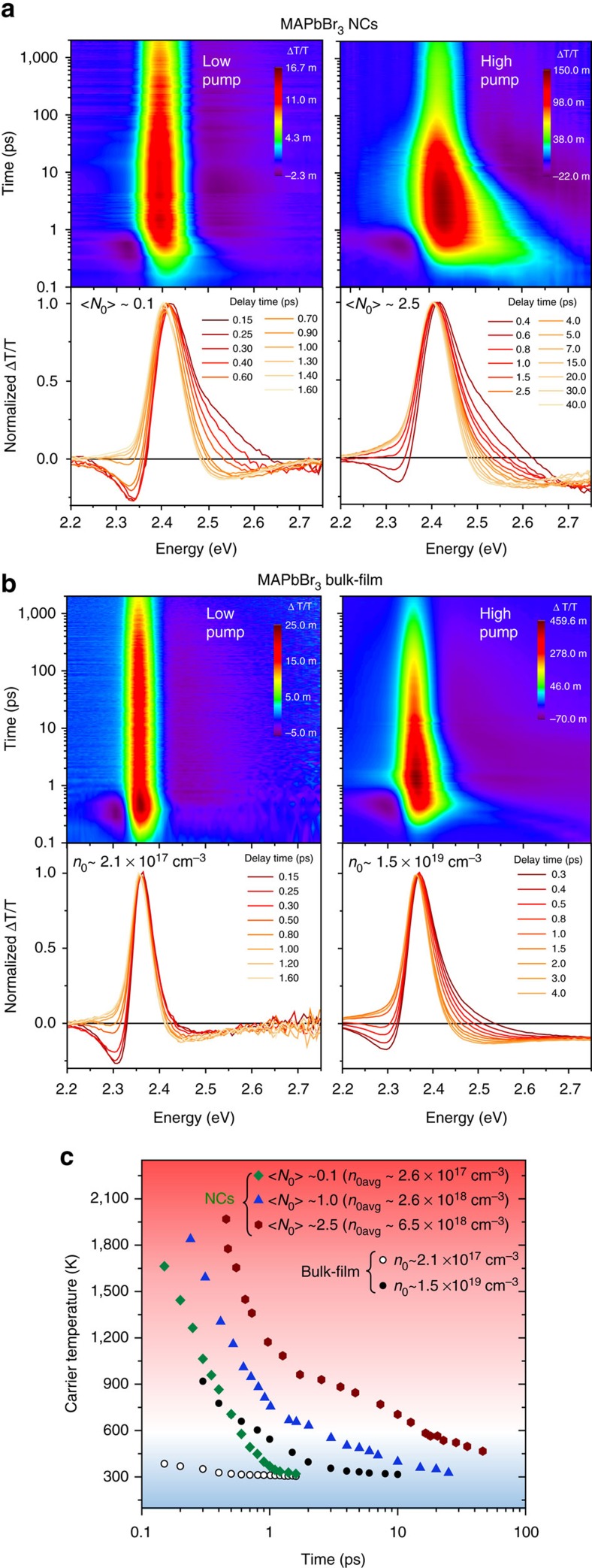
Ultra-slow hot-carrier cooling in perovskites NCs. (**a**) Pseudo colour TA plot (upper panel) and normalized TA spectra (lower panel) for medium MAPbBr_3_ NCs (radii ∼4–5 nm) in solution at low pump fluence (left panel) with initially generated <*N*_0_> ∼0.1 (*n*_0avg_∼2.6 × 10^17^ cm^−3^) and high pump fluence (right panel) with <*N*_0_> ∼2.5 (*n*_0avg_∼6.5 × 10^18^ cm^−3^). (**b**) Pseudo colour representation (upper panel) and normalized TA spectra (lower panel) for MAPbBr_3_ bulk film at low pump fluence (left panel) with initially generated carrier density *n*_0_∼2.1 × 10^17^ cm^3^ and high pump fluence (right panel) with *n*_0_ ∼1.5 × 10^19^ cm^3^. (**c**) Hot-carrier temperature as a function of delay time for the medium MAPbBr_3_ NCs and bulk film with different carrier densities. Photoexcitation energy for **a**,**b** is 3.1 eV.

Figure 1a,b show a comparison of the pseudo colour TA plots and TA spectra of the medium MAPbBr_3_ NCs (radius around 4.5 nm) versus MAPbBr_3_ bulk film at low and high pump fluence, respectively. For both types of samples, the plots/spectra display a prominent photobleaching (PB) peak with a high-energy tail near the bandgap because of the state-filling effects. Similar results were also observed in the small and large NCs (Supplementary Fig. 5). For the bulk sample, the high-energy tails of the PB peak originate from the rapid distribution of initial non-equilibrium carriers into a Fermi-Dirac distribution via elastic scatterings (including electron-hole scattering at low pump fluence^19^ and carrier–carrier scattering at high pump fluence) that can be characterized by a carrier temperature *T*_c_. *T*_c_ can thus be extracted by fitting the high-energy tail of the TA spectra with a simple Maxwell–Boltzmann function of 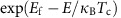, where *κ*_B_ is the Boltzmann's constant and *E*_f_ is the quasi-Fermi energy (see Supplementary Note 2).

For NCs, the discrete energy levels can be approximately treated as a continuum in the case where thermal energy *κ*_B_*T* is much larger than energy level spacing Δ*E*^20^. In contrast to conventional semiconductor NCs with strong confinement, our perovskites NCs are in the weak confinement regime with energy levels that are more closely spaced (see Supplementary Note 1 for the discussion of weak confinement). Thus, in the microscopic picture of a single dot, we expect that the efficient electron-hole scattering (due to the enhanced Coulomb interaction under confined conditions) at low pump fluence^19^ (that is, with one electron-hole pair per NC) and carrier–carrier scattering at high pump fluence can cause the rapid non-thermal energy distribution to evolve into a Fermi-Dirac-like distribution within 150 fs. In the macroscopic picture, our TA spectra are collected from an ensemble of NCs whose size distribution will cause inhomogeneous broadening (that is, overlapping TA spectrum from single NC). All these properties rightly lead to a continuous TA spectrum from the NCs ensemble, to resemble that of the bulk materials. Hence, the high-energy tail of our NCs' TA spectra can also be described by a Maxwell–Boltzmann distribution. The representative fits of the high-energy tails and non-normalized TA spectra are presented in Supplementary Fig. 6. It is evident from both the pseudo colour TA plots and spectra that the high-energy tails (starting at the kink in the spectrum where the exponential decay region begins, see Supplementary Fig. 6) of the PB peak persists much longer for NCs than that of the bulk film, suggesting higher carrier temperatures and slower hot-carrier cooling.

### Slower hot-carrier cooling in perovskites NCs

Figure 1c shows the temporal evolution of *T*_c_ for the medium NCs and bulk film at various pump fluence: initial photoexcited hot-carrier density *n*_0_ (bulk-film) and average generated electron-hole pairs per NC (<*N*_0_>=*Jσ*, where *J* is the pump fluence and *σ* is the absorption cross-section) (see Supplementary Fig. 7 and Supplementary Note 3 for the determination of *σ*). For comparison with bulk films, we also determined the average carrier density per NC volume in NCs, which is defined as 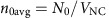, where *V*_NC_ is the NC volume^9^. For NCs, the maximum *T*_c_ at the excitation onset with <*N*_0_> about 0.1 is around 1,700 K, which is about four times higher than that for the bulk-film sample with comparable carrier densities. We attribute the smaller *T*_c_ in the latter to arise from the ultrafast cooling of hot carriers, which had occurred on a timescale much shorter than the temporal resolution of our TA measurements.

It is important to note the complex interplay of the hot-carrier cooling times due to several factors: (i) the pump energy (that is, carriers' excess energy—typically, higher excess energies lead to longer hot carrier lifetimes); (ii) the initial hot-carrier densities (that is, higher carrier densities usually lead to longer hot carrier lifetimes); and (iii) the energy loss rate at a specific hot-carrier temperature (that is, in general, lower hot-carrier temperatures yield smaller energy loss rates). Hence, due care must be taken for a fair comparison of the reported values in the literature. More discussion on hot-carrier lifetime comparison between different materials can be found in Supplementary Note 4. Furthermore, for clearer and easier comparison of hot-carrier cooling lifetimes reported for different materials in the literature, the hot-carrier cooling lifetime in this study is defined as the time interval from pulse excitation till the cooling of hot carriers to 600 K. This temperature is used as the benchmark, because previous theoretical calculations^1^,^21^ have shown that for *T*_c_>600 K (as marked with a red graded background over this region in Fig. 1c), there is still an appreciable hot-carrier conversion efficiency (that is, >40%) over a wide range of absorber bandgaps for photovoltaic applications.

With these considerations in mind, for the control MAPbBr_3_ bulk film, hot-carrier cooling lifetimes ranging from<0.1 to 0.8 ps were obtained for *n*_0_ around 0.21–15 × 10^18^ cm^−3^. These lifetimes are of the same magnitude as those reported for MAPbI_3_ thin films^17^,^18^ excited at similar *n*_0_ and excess energies of 0.7 eV; but is shorter than those for highly excited hot carriers with twice the excess energies (around 1.44 eV)^18^. Notably, our MAPbBr_3_ NCs exhibit hot-carrier cooling lifetimes one to two orders longer than those of the perovskite bulk-film control sample under similar *n*_0avg_ (Supplementary Table 1). For instance, the hot-carrier cooling lifetimes can be as long as about 32 ps (Supplementary Fig. 8) for large NCs with <*N*_0_> around 2.5 (or *n*_0avg_ around 3.5 × 10^18^ cm^−3^). This latter lifetime is about 40 × longer than that for the bulk-film sample, where the latter was excited at almost one order higher carrier density of 15 × 10^18^ cm^−3^. In fact, the hot-carrier cooling lifetimes of our MAPbBr_3_ NCs are much longer than those reported for other semiconductor bulk and nano materials. With reference to Supplementary Table 1, for GaAs thin film, the reported cooling lifetime is about 2 ps with carrier densities around 6.0 × 10^18^ cm^−3^ and excess energies of 1.7 eV; and for CdSe nanorods, the reported cooling lifetime is about 0.8 ps with carrier densities around 5.5 × 10^18^ cm^−3^ and excess energies of 1.1 eV. Our MAPbBr_3_ NCs compares very favourably with much longer lifetimes of 18 ps, excited with much lower excess energies of around 0.7 eV at comparable carrier densities of 6.5 × 10^18^ cm^−3^.

### Intrinsic phonon-bottleneck effect

To discern the slower hot-carrier cooling mechanisms in MAPbBr_3_ NCs, we first examine the relaxation dynamics under low pump excitation. For <*N*_0_> around 0.1, up to 10% of NCs are excited with one *e*–*h* pair based on the Poisson distribution (Supplementary Fig. 7). At such low carrier densities, multi-particle recombination is negligible, which is evident from the absence of any fast decay of band-edge carriers in NCs (Supplementary Fig. 9). Hence, the hot-carrier relaxation mechanism at low pump excitation is therefore representative of the material's intrinsic properties and are not influenced by extrinsic effects such as the multi-particle Auger-recombination, which we will discussed later.

**Figure 2:**
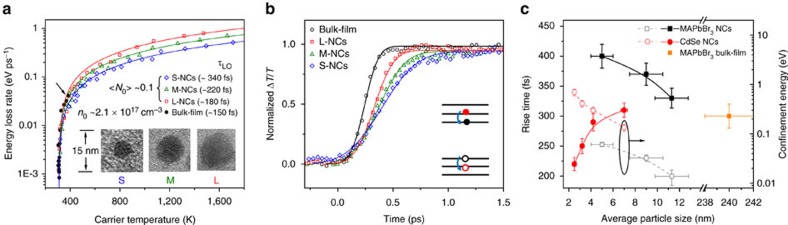
Phonon-bottleneck effect in perovskite NCs at low carrier densities. (**a**) Energy loss rate of hot carriers as a function of carrier temperature *T*_c_ for MAPbBr_3_ NCs with <*N*_0_> ∼0.1 and MAPbBr_3_ bulk film with *n*_0_ ∼2.1 × 10^17^ cm^−3^. Solid lines represent the numerical fits with LO–phonon model. The black arrow indicates the maximum *T*_c_ obtained for the bulk film. Inset shows the representative TEM images of small (S), medium (M) and large (L) perovskites NCs. (**b**) Normalized bleaching dynamics probed at the band-edge for colloidal MAPbBr_3_ NCs and bulk film at low carrier density. Solid lines are the single exponential fits. Inset shows a schematic of the phonon bottleneck induced slow hot-carrier cooling in symmetric conduction and valence bands with discrete energy levels. (**c**) Size dependence of the rise time of band-edge bleachings in MAPbBr_3_ NCs (black solid square), bulk-film (dark yellow solid square) (the particle size is represented by the film thickness) and CdSe NCs (red solid circle). Size dependence of quantum confinement energies in MAPbBr_3_ NCs (black hollow square) and CdSe NCs (red hollow circle). Error bars on the *x* axis represent the size distribution of NCs and on the *y* axis represent uncertainties in the fitting of the rise time and confinement energies. Photoexcitation energy for **a**–**c** is 3.1 eV.

At low pump fluence, *T*_c_ decays faster with increasing NC size (Supplementary Fig. 8). For all three NCs, the energy loss rates per carrier *J*_r_ (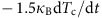) slowly decrease within the range of 0.6–0.3 eV ps^−1^ until *T*_c_ reaches ∼700 K (Fig. 2a), beyond which *J*_r_ falls by several orders of magnitude until *T*_c_ approaches the lattice temperature. Such cooling trend is similar to that for the bulk-film sample as well as in other bulk inorganic semiconductors and nanostructures^10^,^22^,^23^. Here, the initial rapid cooling (that is, higher cooling rate) is attributed to the carrier coupling to longitudinal optical (LO) phonons, which establishes a thermal equilibrium between the LO–phonon population and the hot carriers. Comparing the different NCs, the initial *J*_r_ for small NCs is smaller than the large NCs by a factor of about 2 (indicating a weaker carrier–phonon interaction in the former). The subsequent slower cooling of the hot carriers closer to the band edges (that is, around 300–500 K in Fig. 2a) is determined by the thermal equilibration between LO phonons and acoustic phonons^23^,^24^. Furthermore, from Raman measurement (Supplementary Fig. 10), the available phonon modes for hot-carrier cooling in MAPbBr_3_ NCs are located at around 150 cm^−1^ (assigned to the stretching of the Pb–Br bonds^25^) and 300 cm^−1^ (which could be from the second-order of 150 cm^−1^ and/or the torsional mode of MA cations^25^), respectively.

The energy loss rate was fitted using a LO–phonon interaction model^23^ (see Supplementary Note 5), the fitted *τ*_LO_ (characteristic LO–phonon decay time) increases with reducing NCs dimensionality (see Fig. 2a), which provides direct evidence of the reduction in the optical phonon relaxation by quantum confinement. This is a characteristic of the phonon bottleneck effect, which thus retards the hot-carriers cooling. Although our NCs are in the weak confinement regime with confinement energy around 15–60 meV, several early theoretical papers^26^,^27^,^28^,^29^ had shown that even in this weak confinement regime where the level spacing is only a few meV, the carrier relaxation mediated by phonon interactions can still be dramatically hindered. This is because of restrictions imposed by energy and momentum conservations, and the weak energy dispersion of the LO phonons, which together cause the phonon bottleneck. Moreover, the comparable acoustic phonon temperature (*T*_a_) of ∼310 K for all three NC samples, which is also close to the lattice temperature at room temperature, strongly suggests that the deceleration of hot-carrier cooling is unlikely to be induced by the acoustic phonon bottleneck.

The band-edge bleach buildup approach was also used to elucidate the hot-carrier cooling properties (see Supplementary Note 6). Figure 2b shows the normalized TA spectra of the perovskite samples probed at their band-edge PB peaks following photoexcitation with similar excess energies at low carrier densities. Each buildup process is fitted with a single-exponential growth function to yield a rise time (*τ*_rise_). The rise of the band-edge bleach occurs at sub-picosecond timescale that becomes slower with decreasing NC size, consistent with the smaller *J*_r_ and slower hot-carrier temperature decay (Supplementary Fig. 8) for smaller perovskites NCs. Surprisingly, the trend exhibited by the perovskite NCs is completely opposite to that for CdSe NCs (spanning the strong to weak quantum confinement regimes; Fig. 2c and Supplementary Fig. 11). Furthermore, the perovskite NCs rise times are also much longer. The faster hot-carrier cooling with decreasing CdSe NCs is consistent with previous reports, which is attributed to an Auger-type energy transfer from the hot electrons to the dense hole states^24^. Our results clearly show that such Auger-transfer mechanism present in conventional inorganic semiconductor NCs is naturally suppressed in perovskites NCs. Concerning the schematic energy level diagram of CdSe NCs (Supplementary Fig. 1), one possible reason could be the symmetric energy dispersion and small effective mass for both electrons and holes of perovskite NCs (Supplementary Note 1)^30^,^31^ illustrated in Fig. 2b (inset). Other factors such as surface reconstruction, surface defects, atom fluctuations and so on^7^ could also result in faster hot-carrier cooling with decreasing inorganic semiconductor size (for example, in quantum confined IV–VI semiconductor PbSe with identical and small-electron and hole-effective masses)^32^. The low defect density of our perovskite NCs (consistent with the high photoluminescence (PL) quantum yield of around 80%) could thus be another reason for this behavior (that is, intrinsic phonon bottleneck effect). These insights into the slow hot-carrier cooling in perovskite colloidal NCs (under low pump excitation) challenge the conventional wisdom established for traditional semiconductor NCs.

### Auger-heating effect on hot-carrier cooling

**Figure 3:**
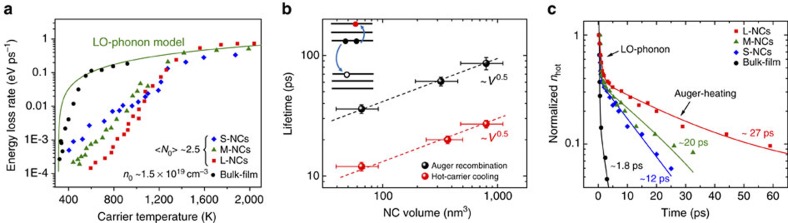
Auger-heating induced slow hot-carrier cooling at high carrier densities. (**a**) Energy loss rate vs carrier temperature *T*_c_ for MAPbBr_3_ NCs with <*N*_0_> around 2.5 and MAPbBr_3_ bulk film with *n*_0_ around 1.5 × 10^19^ cm^-3^. Solid green line represents the LO–phonon model at low carrier densities. (**b**) Auger recombination lifetimes and hot-carrier cooling time versus perovskite NC volume. Black and red dashed lines are guides to the eye showing the scaling of the lifetimes with the square root of NC volume. Inset illustrates the hot-carrier re-excitation by Auger recombination of carriers at band edge (also known as Auger heating). Error bars on the *x* axis account for the size distribution of NCs and on the *y* axis represent uncertainty in the determination of the lifetime. (**c**) Normalized hot-carrier decay at different pump fluences. Solid lines are bi-exponential decay fits. Photoexcitation energy for **a**–**c** is 3.1 eV.

These perovskite NCs also exhibit interesting hot-carrier cooling properties under high pump excitation. Figure 3a shows contrasting trends of energy loss rates versus carrier temperature between the three different sized NCs (at <*N*_0_> around 2.5) and the bulk-film sample (at *n*_0_ around 1.5 × 10^19^ cm^-3^). For both NCs and bulk film, the initial hot-carrier cooling governed by the carrier–LO–phonon interactions is nearly independent of carrier densities. This can be concluded from the following: (i) the almost identical initial fast decay of *T*_c_ at different carrier densities (Fig. 1c) and (ii) the similar initial energy loss rate at high carrier temperatures for both low and high carrier densities (Figs 2a and 3a). For the bulk film, the lengthening of hot-carrier lifetime (Fig. 1c) and the slight deviation of *J*_r_ from the LO–phonon model (Fig. 3a, green line) below 600 K is due to the hot phonon bottleneck effect (commonly observed in bulk inorganic semiconductors and more recently reported for MAPbI_3_ thin films)^18^,^23^,^33^. This is attributed to the reduced decay of LO phonons caused by partial heating of acoustic modes^23^. This assumption is supported by the higher acoustic phonon temperature *T*_a_ of 350 K at the high pump fluence (see Supplementary Fig. 12). However, for NCs, from Fig. 3a, although the *J*_r_ of the NCs (at <*N*_0_> around 2.5) initially follows the LO–phonon model at high carrier temperatures, they deviate greatly from the LO–phonon model as the hot-carrier population cools below 1,500 K. For carrier temperature range below 1,000 K, *J*_r_ reduces drastically by several orders of magnitude when the carrier density is increased from <*N*_0_> around 0.1 (Fig. 2a) to <*N*_0_> around 2.5 (Fig. 3a). For example, at <*N*_0_> around 0.1, *J*_r_ at 700 K is around 0.3 eV ps^−1^, whereas it is around 0.008 eV ps^−1^ at <*N*_0_> around 2.5. Furthermore, *J*_r_ reduces with increasing NC size at carrier temperatures below 1,200 K at <*N*_0_> around 2.5. All these signatures suggest the existence of another slow hot-carrier cooling mechanism that only becomes dominant at high carrier densities, which we posit is the Auger heating mechanism. As it is well-known that Auger recombination is strongly enhanced in confined semiconductor NCs at higher carrier densities due to the increased carrier–carrier interactions^9^. Thus, there is a finite probability for the relaxed hot carrier (at the band edges) to be re-excited to higher energy states through the Auger recombination of carriers at the band edge (Fig. 3b, inset).

Figure 3c shows that the calculated hot-carriers concentration (*n*_hot_(*t*)) for different sized NCs relaxes bi-exponentially with a fast decay occurring within 1 ps and a slower decay of several tens of picoseconds—similar to the behaviour of the hot-carrier temperatures (Fig. 1c and Supplementary Fig. 8). The fast decay is attributed to the carrier–LO–phonon interactions. In addition, the fitted slow decay lifetimes of *n*_hot_(*t*) are well-matched with the one-third relation of their Auger lifetime *τ*_Aug_ (that is, 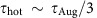; Fig. 3b and Supplementary Note 7). For example, the slower decay lifetime for the small NCs is fitted to be about 12 ps, which is very close to one-third of its *τ*_Aug_ of 38 ps. The excellent agreement between the experimental data and our simple model that includes Auger effects (see Supplementary Note 7) strongly substantiates the dominant Auger heating contribution in further retarding the hot-carrier cooling at high carrier densities. Given that 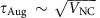 and the slow hot-carrier lifetimes 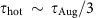, Auger-induced hot-carrier cooling lifetime is sublinearly dependent on the NC volume (Fig. 3b). Although Auger heating causes a slowdown of the hot-carrier cooling rate favourable for hot-carrier extraction, it should also be noted that Auger effects conversely reduce the carrier densities. It is therefore necessary to balance the hot-carrier lifetime and carrier losses in the application of concentrator-type hot-carrier solar cells at high pump fluence.

### Efficient hot-electrons extraction

**Figure 4:**
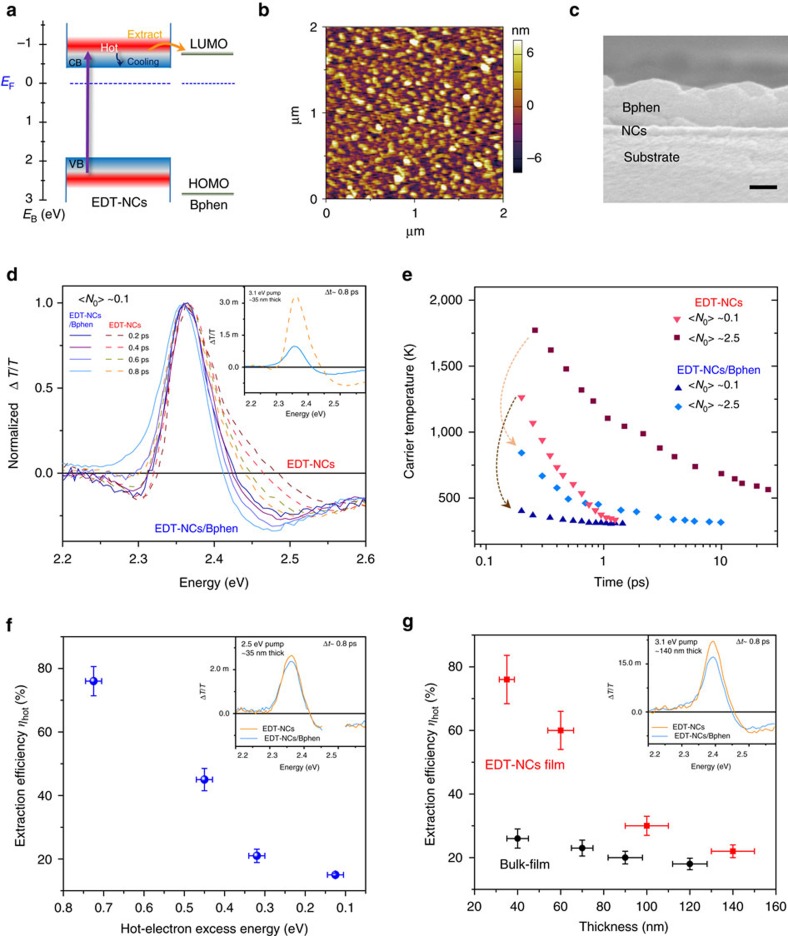
Efficient hot-carrier extraction from perovskites NCs. (**a**) Flat-band energy level diagram for illustration of the hot-electron extraction from perovskites NCs to Bphen with competing hot-electron cooling pathways. Conduction band minimum (CBM) (or LUMO levels) and valence band minimum (VBM) (or highest occupied molecular orbital (HOMO) levels) of NCs (or Bphen) were determined from UPS and ultraviolet–visible measurements. (**b**) AFM image of EDT-NC film. (**c**) Cross-sectional SEM image of EDT-NCs/Bphen bilayer. Scale bar, 100 nm. (**d**) Normalized TA spectra for about 35 nm-thick EDT-NCs film with/without Bphen following 3.1 eV photoexcitation with <*N*_0_> around 0.1. Inset shows the un-normalized TA spectra at 0.8 ps. (**e**) Hot-carrier temperature as a function of delay time for EDT-NCs film and EDT-NCs/Bphen bilayer at different pump fluences. Dotted arrows show the decrease of the initial hot-carrier temperatures after adding the Bphen layer, indicating effective hot-electron extraction. (**f**) Pump energy dependence of the hot-electron extraction efficiencies in about 35 nm-thick EDT-NCs/Bphen bilayer. Inset shows the un-normalized TA spectra at 0.8 ps following 2.5 eV photoexcitation with <*N*_0_> around 0.1 for about35 nm thick EDT-NCs film with/without Bphen. (**g**) Perovskite film thickness dependence of the hot-electron extraction efficiencies upon 3.1 eV pump energy excitation for EDT-NCs/Bphen and bulk-film/Bphen. Inset shows the un-normalized TA spectra at 0.8 ps following 3.1 eV photoexcitation with <*N*_0_> around 0.1 for about 140 nm-thick EDT-NCs film with/without Bphen. Error bars on the *x* axis represent the uncertainties in the determination of (**f**) excess energies and (**g**) sample thickness and on the *y* axis represent uncertainties in the determination of extraction efficiencies.

Apart from slow hot-carrier cooling, the feasibility of efficient hot-carrier extraction is another challenging issue for hot-carrier solar cells. Such extraction must be very fast to limit energy loss, where the competition is between extraction rate and cooling rate rather than recombination rate^21^. Considering the estimated hot-carrier diffusion length of around 16–90 nm (depending on hot-carrier lifetime and diffusion coefficient) in MAPbBr_3_ film (Supplementary Note 8), it is therefore technically feasible to extract hot carriers. Here, efficient hot-electron extraction from 1,2-ethanedithiol (EDT)-treated MAPbBr_3_ NCs (EDT-NCs) to 4,7-diphenyl-1,10-phenanthroline (Bphen) is demonstrated. Bphen is selected as the hot-electron extraction material, because this molecular semiconductor has a high electron mobility^34^ and possess a higher lowest unoccupied molecular orbital (LUMO) than the conduction band minimum of our EDT-treated NCs (see Supplementary Fig. 13 for ultraviolet photoelectron spectroscopy (UPS) measurements), implying only hot-carriers with sufficient excess energies above band-edge can be injected into Bphen (Fig. 4a). Bphen also possesses a narrow electron bandwidth^35^, which allows it to approximate the energy selective contact required in hot-carrier solar cells^36^. EDT treatment is used to substitute the long and highly insulating oleic acid and oleylamine ligands that is present on the as-prepared NC surfaces with thiolate^37^ (see attenuated total reflection—Fourier transform infrared (FTIR) and X-ray photoelectron spectroscopy (XPS) measurements in Supplementary Fig. 14 and Supplementary Note 9) for more effective electronic coupling with Bphen and within NCs films^38^ (evident from the closer NCs packing after treatment as shown in TEM images in Supplementary Fig. 15).

Hot-electron extraction from spin-coated EDT-NCs thin film (see atomic force microscopy (AFM) and SEM images in Fig. 4b,c) by Bphen is validated by the clear reduction of the high-energy tails of TA spectra for the EDT-NCs/Bphen bilayers that occurs instantaneously (see Fig. 4d, pseudo colour TA spectra in Supplementary Fig. 16 and Supplementary Note 10). For around 35 nm-thick EDT-NCs film, after adding Bphen layer, the initial *T*_c_ cooled from around 1,300 to 450 K at low pump intensity and from around 1,800 to 800 K at high pump intensity within about 200 fs after photoexcitation (Fig. 4e), indicating that the carriers with higher energies and temperature are injected into Bphen. From the conduction band minimum and LUMO offset between EDT-NCs and Bphen, hot carriers with excess energy larger than 0.2±0.1 eV are extracted (Supplementary Fig. 17). Considering the increased diffusion coefficient for hot carriers (see Supplementary Note 8) and ultrafast hot-carrier hopping between NCs (within several tens of femtoseconds)^39^, we believe that the hot electrons are likely to be injected into Bphen through electron diffusion inside the NC and hopping at NC interfaces. The driving force of hot-electron transfer is the energy difference between the hot-carrier energy and the LUMO energy with respect to the Fermi energy as shown in Fig. 4a. A large density of states is typical for organic molecules. The highly efficient hot-carrier transfer is thus attributed to the high density of acceptor states in LUMO levels of Bphen together with the strong electronic coupling between Bphen and NCs. Additional control experiments to prove that Bphen is the only pathway for extraction of hot carriers from NCs in EDT-NCs/Bphen can be found in Supplementary Note 11. Notably, we also observed similar near-infrared (NIR) photoinduced absorption signals for EDT-NCs/Bphen (in which mainly NCs were selectively excited) and the Bphen (above-bandgap excitation) (see Supplementary Fig. 18 and Supplementary Note 12), which could arise from radical anion absorption and/or excited singlet absorption in Bphen. Thus, we attribute the photoinduced absorption signal in EDT-NCs/Bphen to the population of transferred hot carriers from NCs to the LUMO of Bphen. Nonetheless, there is also the possibility of an alternative explanation of excited singlet absorption induced by hot-state energy transfer from NCs to Bphen.

The efficiency of hot-electron extraction (*η*_hot_) is estimated based on the percentage reduction of the band-edge PB intensities at around 0.8 ps after adding Bphen (as when the hot electrons are relaxed to the band edges, the reduced band-edge bleaching intensity can then be attributed to hot-carriers extraction). Calculated *η*_hot_ for around 35 nm-thick EDT-NCs/Bphen bilayer is about 72% and 58% at <*N*_0_> are around 0.1 and 2.5 pump intensities, respectively. The reduced multi-hot-electron injection efficiency at higher pump fluence may be due to the increased back-electron transfer from Bphen to the NCs with the estimated back-electron transfer time of about 80 ps (Supplementary Fig. 19 and Supplementary Note 13). Considering the voids in the NCs film (Fig. 4b, AFM image), some Bphen molecules could penetrate the upper layer of NCs film, which could further enhance the hot-carrier extraction efficiency by increasing the NC/Bphen interfaces in the thinner NCs film/Bphen bilayers. Furthermore, we also found that moderate post-heating (for example, at 70 °C for 5 min) of EDT-NCs not only further enhanced the electronic coupling^40^ (see XPS spectra in Supplementary Fig. 14 and Supplementary Note 9) and *η*_hot_ to about 83% (Supplementary Fig. 20a), but also increased the Fermi-level of NCs relative to its valence band minimum (Supplementary Fig. 17) (possibly due to the sulfur doping during annealing). This enables the extraction of hot carriers with even higher excess energies above the band edges (up to 0.5±0.1 eV) with *T*_c_ cooling from around 1,300 to 400 K (Supplementary Fig. 20b).

Further proof of hot-electrons injection into Bphen is revealed by the pump-energy dependent *η*_hot_. As shown in Fig. 4f, *η*_hot_ decreases from ∼72% to 15% with a reduction of the excess hot-carrier energies from about 0.7 to 0.1 eV (using above bandedge excitation with pump energy from 3.1 to 2.5 eV). These results validate that only hot carriers with sufficient excess energies can be injected into Bphen (consistent with the energy level diagram in Fig. 4a). They also demonstrate the high selectivity of using Bphen with narrow LUMO, to extract the hot electrons. Importantly, when the thickness of NCs film increases from ∼35 to ∼140 nm (Supplementary Fig. 21), 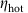 drops dramatically from about 72 to 20% following 3.1 eV photoexcitation (Fig. 4g). The reduced hot-electron extraction should be caused by the limited hot-electron diffusion/hopping range inside the NCs films. Comparatively, owing to the initial rapid hot-carrier cooling, the *η*_hot_ of bulk-film/Bphen with thickness of ∼240 nm is ∼16 % with changing of *T*_c_ only from about 450 to 380 K under similar photoexcitation conditions (Supplementary Fig. 20c,d). Even when the bulk-film thickness is reduced to about 40 nm, 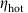 is still much smaller than EDT-NCs film.

## Discussion

We uncovered approximately two orders slower hot-carrier cooling times and about four times larger hot-carrier temperatures in colloidal MAPbBr_3_ NCs as compared with perovskites bulk films under similar photoexcitation conditions. Under low pump fluence, hot-carrier cooling in NCs is mediated by the phonon bottleneck effect, which is surprisingly slower in smaller NCs (contrasting with conventional NCs). This finding contravenes the conventional understanding in traditional colloidal semiconductor NCs that intraband Auger effects is more dominant with decreasing dimensionality, resulting in the breach of the phonon bottleneck. At high pump fluence, Auger heating dominates the hot-carrier cooling rate, which is slower in larger NCs (previously unobserved in conventional NCs). Importantly, the augmented slow hot-carrier cooling in these colloidal perovskite NCs enables efficient hot-carrier extraction. We demonstrated that the hot electrons with up to around 0.6 eV excess energy can be efficiently injected (up to about 83%) from surface-treated MAPbBr_3_ NCs films into electron acceptor layers with an injection time of around 0.2 ps.

Our findings of the hot-carrier properties in perovskites NCs enable fresh opportunities for extremely thin absorber (ETA) and concentrator-type hot-carrier solar cells. For the former, ETA-solar cells are conceptually close to dye-sensitized heterojunctions^41^. The molecular dye is replaced by an extremely thin (around tens of nanometres) semiconductor absorber layer. By nanostructuring the electrodes (for example, using highly porous TiO_2_ scaffold, ZnO nanowire arrays and so on), the effective area covered by the thin absorber can be increased by several orders of magnitude due to the surface enlargement and multiple scattering^41^. Most importantly, the ETA layer is extremely beneficial for hot-carrier extractions owing to the shorter transport path length for hot carriers. For the later, the illumination power in the concentrator-type solar cells can be increased to 1,000 suns, much larger than the 1 sun intensity in typical cells; the Auger-heating-induced slower hot-carrier cooling in perovskite NCs would also be applicable.

## Methods

### Size-varied perovskite NC synthesis

MAPbBr_3_ NCs were synthesized by the ligand-assisted re-precipitation method as reported by Zhang *et al*.^42^. At first, in a glass vial, 0.16 mmol of methylammonium bromide (MABr) and 0.2 mmol of lead bromide (PbBr_2_) were mixed in 5 ml dimethylformamide (DMF) solution. Later, 50 μl of oleylamine and 0.5 ml oleic acid were also mixed in the DMF solution to form the final precursor solution. Another round-bottom flask containing 5 ml of toluene was preheated to 60 °C in an oil bath. Then 250 μl of as-prepared precursor solution was swiftly injected into hot toluene solution under vigorous stirring condition. The solution immediately turned green, confirming the formation of MAPbBr_3_ NCs. The reaction was continued for another 5 min and stopped by cooling in a water bath. The reaction solution was transferred into a centrifuge tube and centrifuged at the desired speed for different sized spherical-shaped MAPbBr_3_ NCs. The precipitation of MAPbBr_3_ NCs was re-dissolved in toluene solution for further studies. For small-, medium- and large-sized NCs, the precipitate was separated by using centrifuge speed of 12,000, 8,000 and 4,000 r.p.m., respectively. The mean diameter are about 4.9, 8.9 and 11.6 nm for small-, medium- and large-sized NCs, respectively (Supplementary Fig. 2).

### MAPbBr_3_ bulk-film fabrication

A solution containing 0.6 M MAPbBr_3_ in DMF was spin-coated (5,000 r.p.m., 12 s) on quartz substrates. During spin-coating, few drops of toluene were added to the film at 3 s after the beginning of spinning. The film was then dried in room temperature for 30 min and annealed at 70 °C for 5 min. All the film deposition and annealing was done in N_2_-filled glove box. The grain size of bulk-film is larger than ∼1 μm and the thickness is around 240 nm (Supplementary Fig. 3).

### MAPbBr_3_ NCs and EDT-NC film fabrications

MAPbBr_3_ NC film and EDT-treated NCs were grown by a layer-by-layer spin-coating processing method. All the spin-coating steps were set at 1,000 r.p.m. and spin time was fixed for 30 s. To prepare the NC film, NCs in toluene (10 mg ml^−1^) was spin-coated on glass substrates for two layers. For the EDT treatment of NCs film, the growth of each layer of EDT-treated NCs film consisted of three steps: (1) spin-coating of NC solution on top of substrate; (2) cover the NC film with 0.2 M EDT solution in 2-Propanol and wait for 30 s and then spin coat; (3) dropping of anhydrous toluene on film and followed by spin-coating to clean the remaining long-chained ligands. The above process was repeated for two to ten times, to obtain the NC film with different thickness. For post-annealed samples, the annealing was performed at 70 °C for 5 min. All processing was performed in a N_2_-filled glove box.

### Bphen thin-film fabrication

Bathophenanthroline (or Bphen) was deposited through a thermal evaporation method under a pressure of 10^−6^ torr. Bphen was deposited on spin-coated non-annealed or annealed perovskites NCs films at a rate of 0.1–0.2 nm s^−1^.

### CdSe NCs

CdSe NCs dispersed in toluene were purchased from Sigma-Aldrich Co. LLC.

### TA measurements

TA measurements in the time range of femtosecond to nanosecond were performed using a Helios spectrometer (Ultrafast Systems, LLC). The pump pulse was either generated from an optical parametric amplifier (Coherent OPerA Solo or Light Conversion TOPAS-C) that was pumped by a 1 KHz regenerative amplifier (that is, Coherent Libra (50 fs, 1 KHz, 800 nm) or Coherent Legend (150 fs, 1 KHz, 800 nm)) or by frequency doubling the 800 nm fundamental regenerative amplifier output with a BBO crystal to obtain 400 nm pulses. Both systems were seeded by mode-locked Ti-sapphire oscillators (Coherent Vitesse, 80 MHz). The white light continuum probe beam (in the range from 400 to 1,500 nm) was generated by focusing a small portion (∼10 μJ) of the regenerative amplifier's fundamental 800 nm laser pulses into either a 2 mm sapphire crystal (for visible range) or a 1 cm sapphire crystal (for NIR range). The probe beam was collected using a CMOS sensor for ultraviolet–visible region and InGaAs diode array sensor for NIR region. The samples were kept in a N_2_-filled chamber at room temperature during measurements. For the hot-carrier extraction measurements, the pump beam excited the samples from the side of Bphen based on the sample structure of Bphen/perovskite/glass substrate.

### PL and time-resolved PL measurements

Steady-state PL spectra were collected in a conventional backscattering geometry and detected by a charge-coupled device array (Princeton Instruments, Pixis) coupled to a monochromator (Acton, Spectra Pro). The temporal evolution of PL was resolved by an Optronis Optoscope streak camera system. The excitation source is the same regenerative amplifier (Coherent Libra) and optical parametric amplifier (Coherent OPerA Solo) described above. All the above measurements were performed at room temperature.

### Materials characterization

The shape and size of the NCs were determined by TEM (JEOL JEM-2010). The surface morphology of the perovskite NC films was recorded by AFM (Asylum Research MFP-3D) with a silicon cantilever operating in tapping force mode. The morphology and thickness of samples were characterized by SEM (JEOL, JSM-7600F).

UPS was used to investigate the interfacial energy level alignment of the valence occupied states. The spectra collection was performed with the same instrument as that in XPS. The excitation source is He-I (*h*=21.2 eV) with lamp power at 50 W. Photoelectrons were collected at surface normal using CAE mode with 2.00 eV pass energy with the samples biased at −10 V. XPS was performed to analyse the composition of samples. Samples were transferred to an ultra-high vacuum analysis chamber from the glove box through an air-tight sample transfer containment. The pressure of the ultra-high vacuum chamber was held under 1 × 10^−9^ torr. Al Kα (*hν*=1486.6 eV) photon source at 200 W were used to excited the sample, whereas spectra collection was performed through a hemispherical electron energy analyser (Omicron EA-125). The measurements were performed at room temperature, with photoelectrons collected along surface normal direction.

The crystal structures were analysed by powder X-ray diffraction (Bruker D8 Advance). The absorption spectra were recorded using a ultraviolet–visible spectrometer (SHIMADZU UV-3600 UV–VIS–NIR Spectrophotometer) with an integrating sphere (ISR-3100). FTIR spectra of the all samples were measured by a Frontier FTIR/NIR spectrometer (PerkinElmer, Waltham, MA, USA) equipped with a universal attenuated total reflection sampling accessory (PerkinElmer). Raman spectra were recorded with a WITec Raman microscope (WITec GmbH, Ulm, Germany) using a 633 nm HeNe laser as the excitation source.

### Data availability

The data that support the findings of this study are available from the corresponding author upon reasonable request.

## Additional information

**How to cite this article:** Li, M. *et al*. Slow cooling and highly efficient extraction of hot carriers in colloidal perovskite nanocrystals. *Nat. Commun.* **8,** 14350 doi: 10.1038/ncomms14350 (2017).

**Publisher's note**: Springer Nature remains neutral with regard to jurisdictional claims in published maps and institutional affiliations.

## Supplementary Material

Supplementary Information Supplementary Figures, Supplementary Table, Supplementary Notes and Supplementary References
