# Corrigendum: Slow cooling and highly efficient extraction of hot carriers in colloidal perovskite nanocrystals

**DOI:** 10.1038/ncomms15299

**Published:** 2017-05-25

**Authors:** Mingjie Li, Saikat Bhaumik, Teck Wee Goh, Muduli Subas Kumar, Natalia Yantara, Michael Grätzel, Subodh Mhaisalkar, Nripan Mathews, Tze Chien Sum

Nature Communications 8: Article number: 14350; DOI: 10.1038/ncomms14350 (2017); Published: 02 08 2017; Updated: 05 25 2017

The original version of this Article contained a typographical error in the first sentence of the abstract in which ‘Shockley-Queisser' was incorrectly given as ‘Schottky-Queisser'. This has now been corrected in both the PDF and HTML versions of the Article.

