# Supplementary material for: Slow cooling and highly efficient extraction of hot carriers in colloidal perovskite nanocrystals
Source: Nat Commun. 2017 Feb 8;8:14350. doi: 10.1038/ncomms14350 (PMC5309769; doi:10.1038/ncomms14350)
Supplement: Supplementary Information — Supplementary Figures, Supplementary Table, Supplementary Notes and Supplementary References [file ncomms14350-s1.pdf]

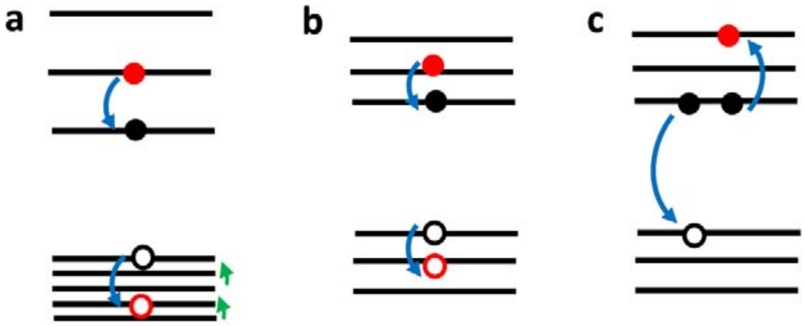

Supplementary Fig. 1 | Schematic hot-carrier cooling with Auger processes in semiconductor nanocrystals. (**a**) Hot-carrier cooling via intraband Auger-type energy transfer. A hot electron (red dot) can be cooled by Auger-type energy transfer to densely spaced hole states (*e.g.*, CdSe NCs), then the hot holes (red circle) can relax rapidly via a cascade of single phonon emissions (green arrows). (**b**) Phonon bottleneck effect induced slow hot-carrier cooling in symmetric conduction and valence bands with discrete energy levels. (**c**) Hot-carrier re-excitation by interband Auger-recombination of carriers at band edges, also called Auger-heating.

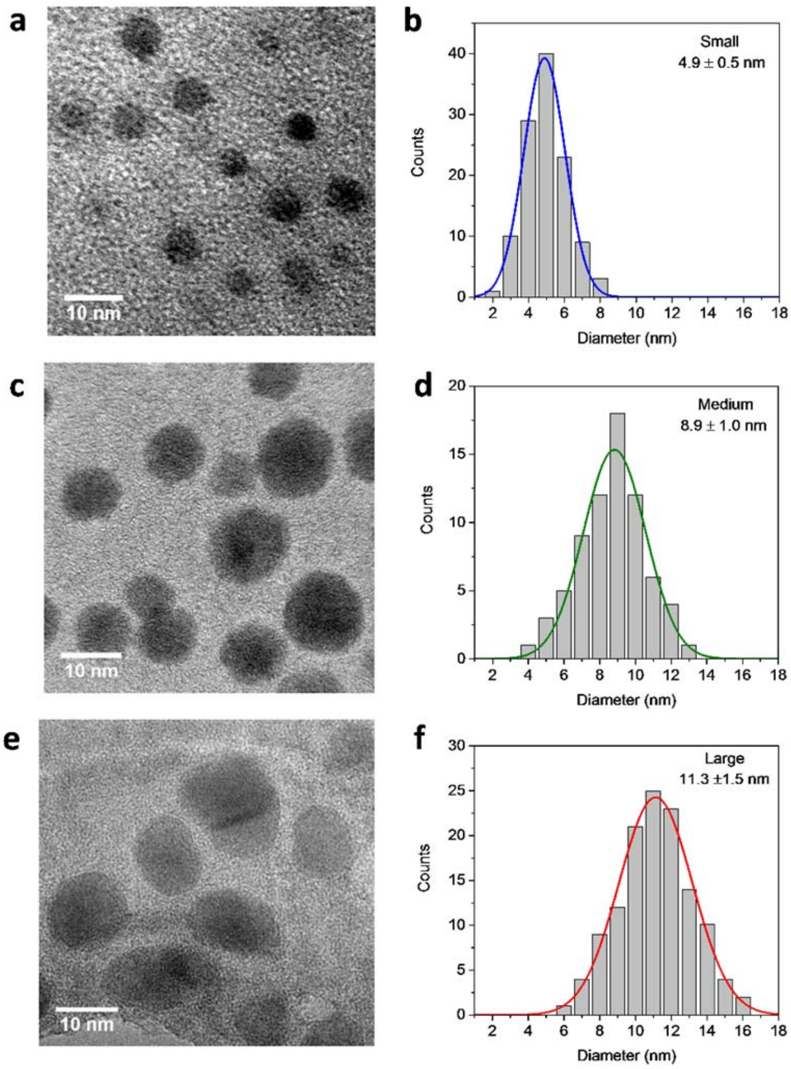

Supplementary Fig. 2 | Representative TEM images of $MAPbBr_3$ NCs with relatively (**a**) small, (**c**) medium and (**e**) large sizes and the size histograms are shown on their right. The size distribution was modeled with a Gaussian distribution.

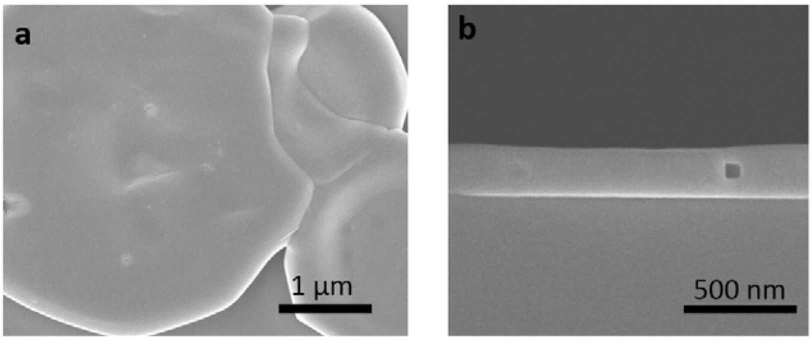

Supplementary Fig. 3 | (**a**) Top- and (**b**) side-view SEM images of $MAPbBr_3$ bulk-film.

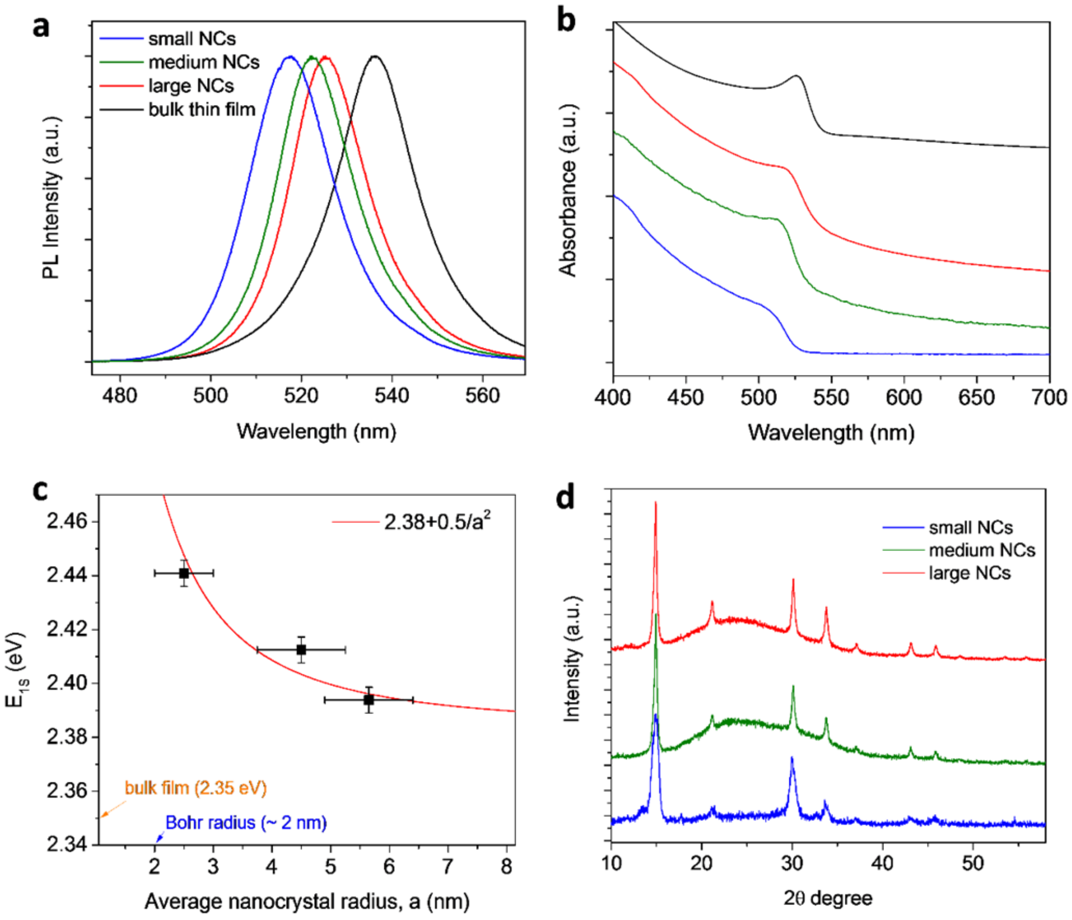

Supplementary Fig. 4 | Room temperature (**a**) PL and (**b**) UV-vis absorption spectra of $MAPbBr_3$ NCs dispersed in toluene and the bulk-film counterpart. (**c**) The energy of the 1s exciton of our $MAPbBr_3$ NCs as a function of radius. The red line is the fitted curve according to equation S1. See Supplementary Note 1 for the discussions. (**d**) XRD patterns of three different sized $MAPbBr_3$ NCs.

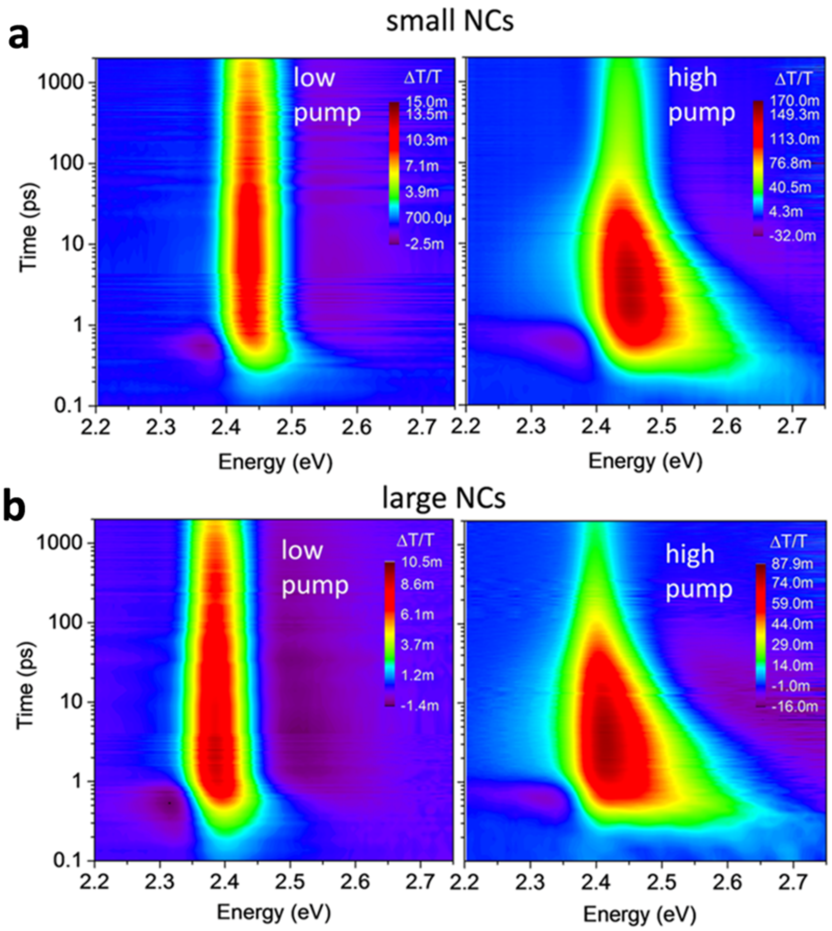

Supplementary Fig. 5 | Pseudocolor TA spectra for (**a**) small and (**b**) large sized $MAPbBr_3$ NCs in solution at low pump fluence (left panel) with initially generated $<N_0>$ ~0.1 and high pump fluence (right panel) with $<N_0>$ ~2.5 following 3.1 eV photoexcitation.

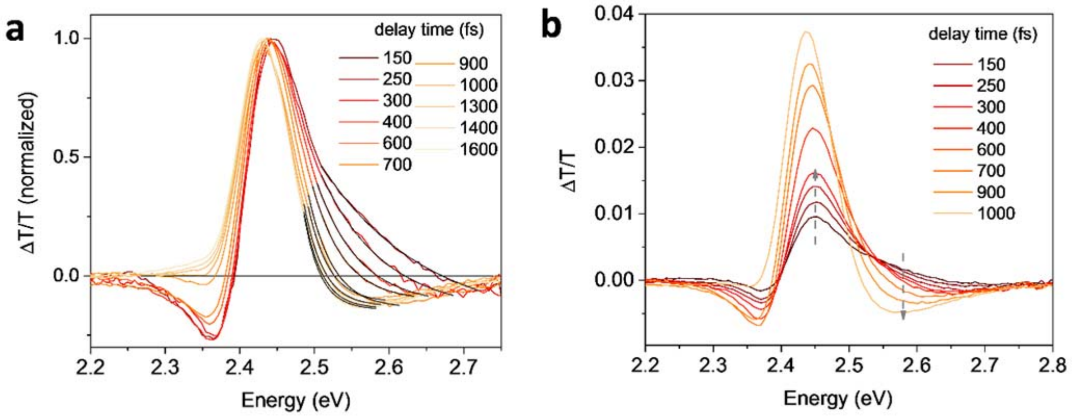

Supplementary Fig. 6 | (**a**) Normalized TA spectra at different short time delays of medium sized $MAPbBr_3$ NCs in toluene with $<N_0>\sim0.1$ (following 3.1 eV photoexcitation), the solid black lines fits to the high-energy tails using the Maxwell-Boltzmann distribution function. (**b**) Un-normalized TA spectra of (a).

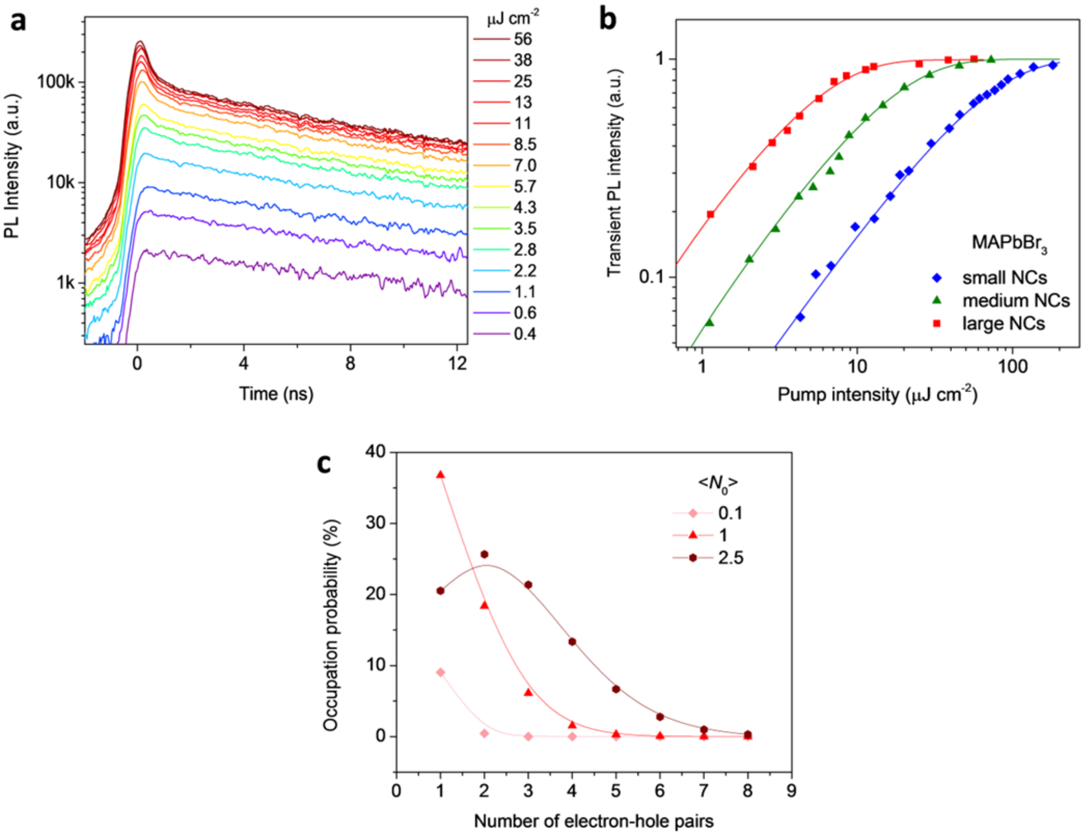

Supplementary Fig. 7 | (**a**) Pump fluence dependent time-resolved PL of the medium $MAPbBr_3$ NCs under 3.1 eV photoexcitation. (**b**) Normalized PL intensities of three different sized $MAPbBr_3$ NCs as a function of pump fluence measured at time $\Delta t = 4$ ns. (**c**) Occupation probability (%) as a function of the number of e-h pairs per NC at different average e-h pairs per NC. See Supplementary Note 3 for discussions.

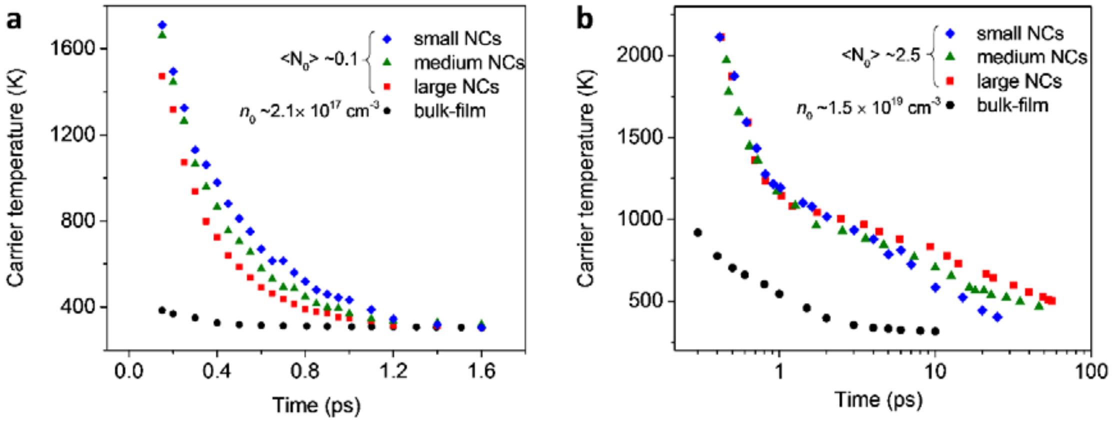

Supplementary Fig. 8 | Hot-carrier temperatures as a function of time delay for three different sized $MAPbBr_3$ NCs and bulk-film (**a**) at low pump fluence (corresponding to $<N_0>$ ~0.1 in NCs and n_0 ~2.1 × 10^{17} cm^3 in bulk-film) and (**b**) at high pump fluence (corresponding to $<N_0>$ ~2.5 in NCs and n_0 ~1.5 × 10^{19} cm^3 in bulk-film) following 3.1 eV photoexcitation.

a
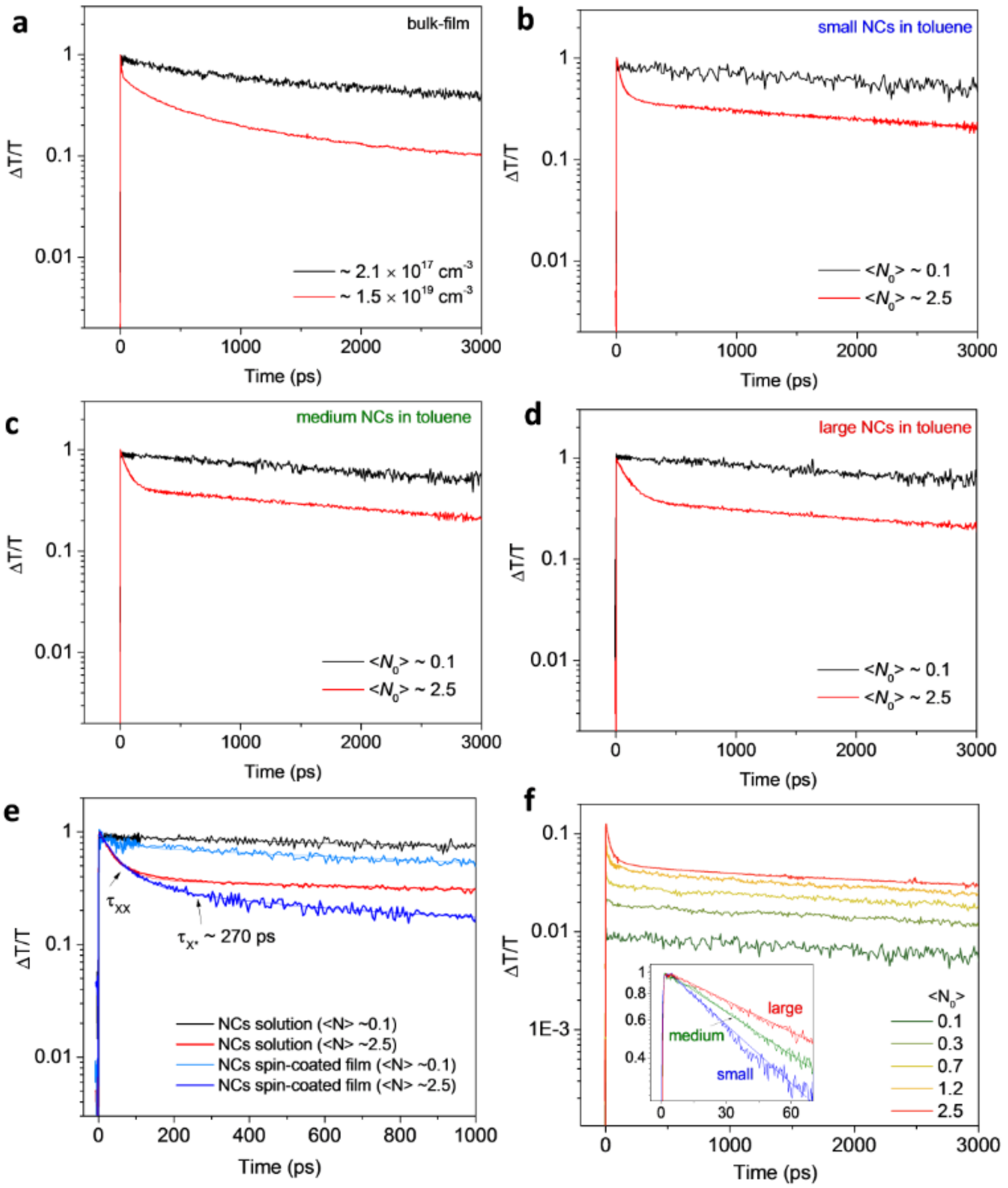

Supplementary Fig. 9 | Normalized photobleaching dynamics probed at the band edges of $MAPbBr_3$ (**a**) bulk-film, (**b**) small (**c**) medium and (**d**) large NCs in solution with high and low pump fluence respectively. (**e**) Comparison of TA dynamics for medium NCs in solution and spin-coated NCs film. (**f**) Pump-fluence-dependent bleaching dynamics probed at the band edge of small $MAPbBr_3$ NCs in solution. Inset: the extracted Auger recombination component after subtracting the single-exciton decay at longer time. Photoexcitation energy: 3.1 eV.

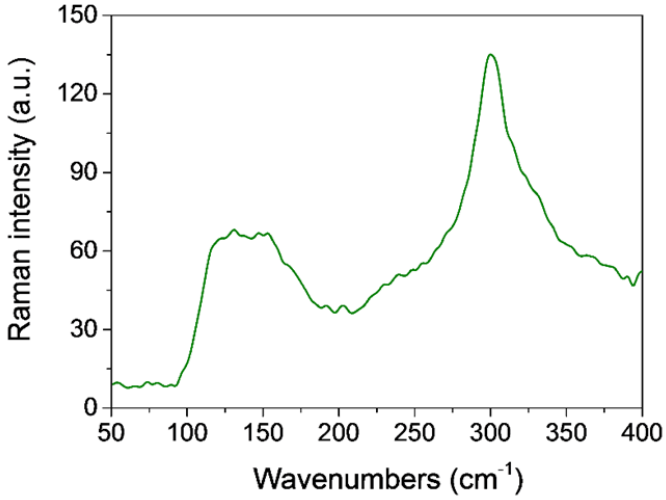

Supplementary Fig. 10 | Room temperature Raman spectrum of as-prepared $MAPbBr_3$ NCs drop-cast on glass substrate, where the peaks originate from LO phonons.

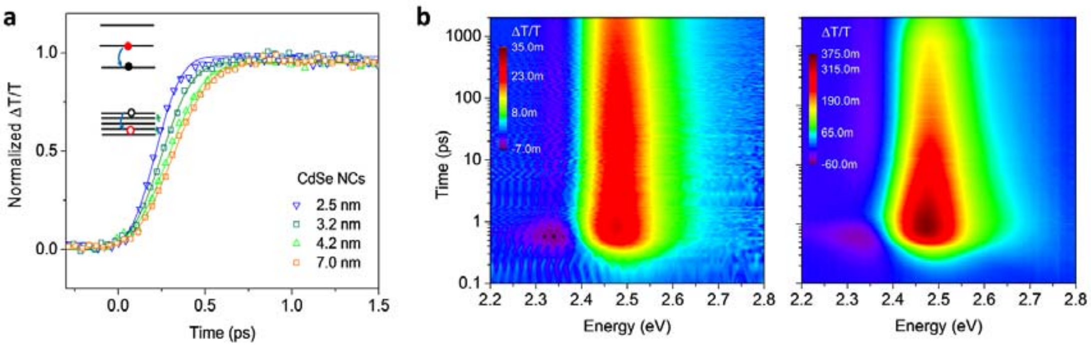

Supplementary Fig. 11 | (a) Normalized bleaching dynamics probed at the band edge for colloidal CdSe NCs with different diameters (shown in legend) at low pump fluence. Solid lines are the single exponential growth fitting curves. The inset schematically shows the hot-carrier cooling process via Auger-type energy transfer. Pseudocolor TA spectra for CdSe NCs (~ 2.5 nm in diameter) at (**b**) low pump fluence with initially generated $<N_0>$ ~0.1 (left) and high pump fluence with $<N_0>$ ~2.5 (right). Phtotoexcitation energy: 3.1 eV.

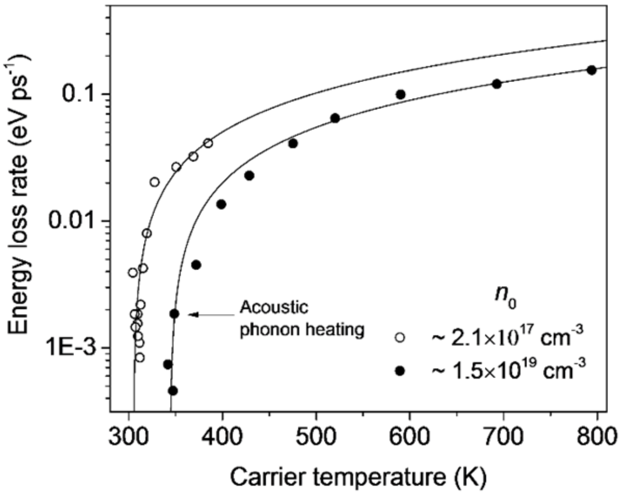

Supplementary Fig. 12 | Energy loss rate of hot-carriers as a function of carrier temperature *T*c for $MAPbBr_3$ bulk-film at low and high carrier densities. Solid lines represent the fits numerically fitted with equation S3. The fitted LO-phonon lifetime $\boldsymbol{\tau_{LO}}$ and acoustic temperature T_a are 150 ± 20, 280 ± 20 fs, and 305 ± 10 and 350 ± 10 K with low and high carrier densities, respectively.

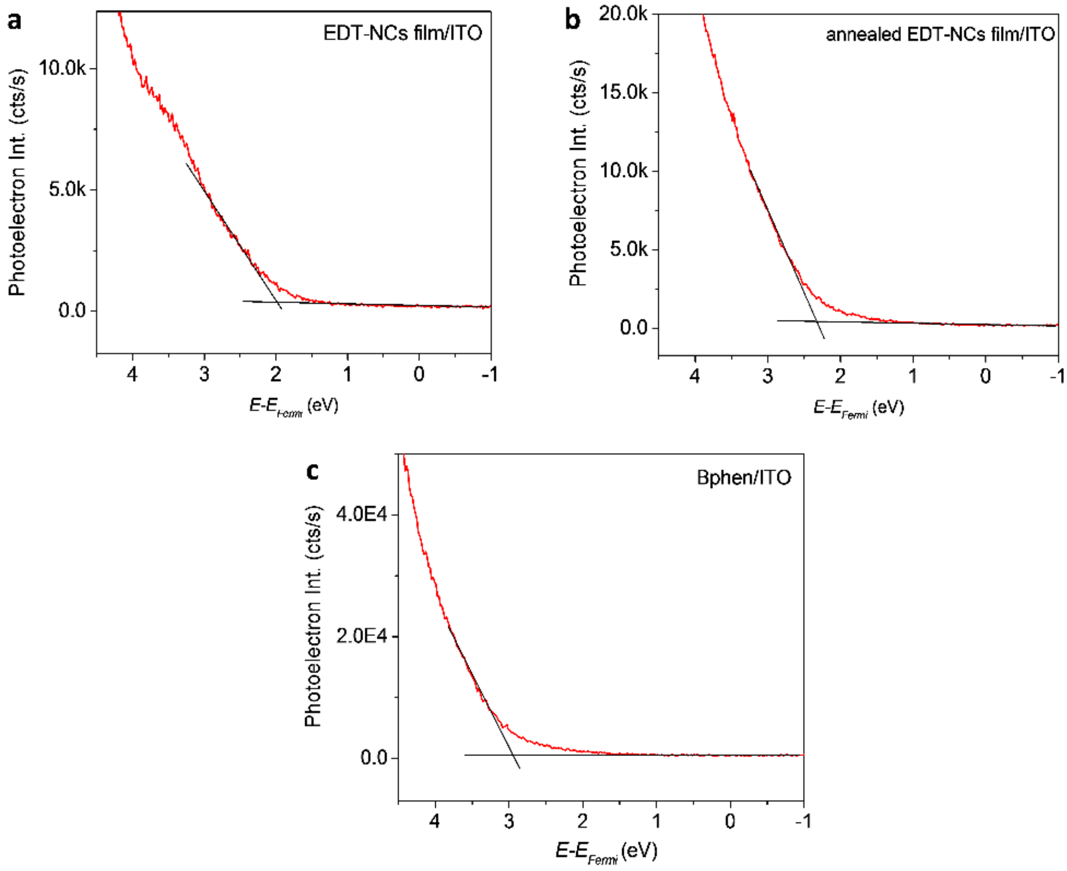

Supplementary Fig. 13 | UPS spectrum of (**a**) EDT-treated and (**b**) post-annealed EDT-treated $MAPbBr_3$ NCs films and (**c**) Bphen film on ITO substrates. The valence band maximum (VBM) is determined by linear extrapolation of the leading edge of the valence band to the background intensity, which is 1.9 ± 0.1, 2.3 ± 0.1 and 2.9 ± 0.1 eV for (a) – (c), respectively.

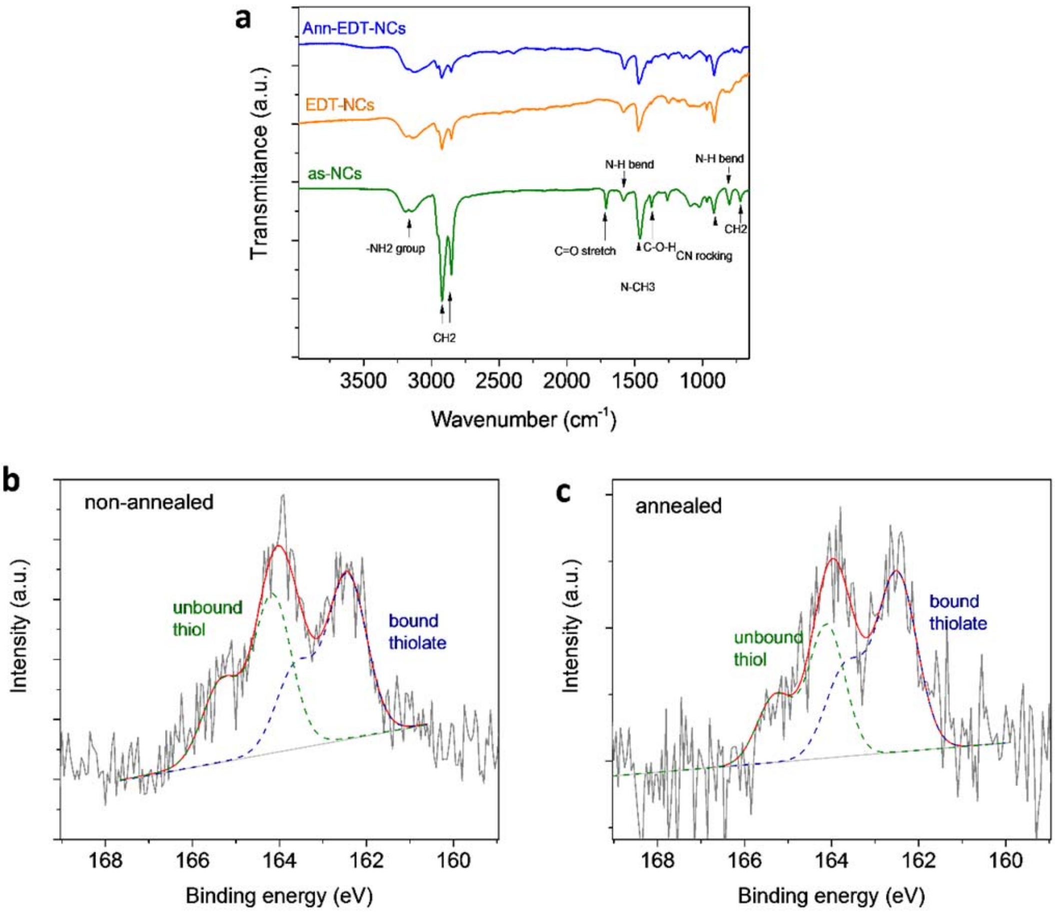

Supplementary Fig. 14 | (**a**) ATR-FTIR spectra of as-prepared $MAPbBr_3$ NCs (green), EDT-treated NCs (orange) and 70 °C annealed EDT-NCs (blue). XPS sulfur (S) 2p spectra of (**b**) non-annealed and (**c**) 70 ^{o}C post-annealed EDT-treated NCs film. S 2p can be deconvolved into unbound thiol (green dashes) and bound thiolate (blue dashes) on the NC surface. See Supplementary Note 9 for discussions.

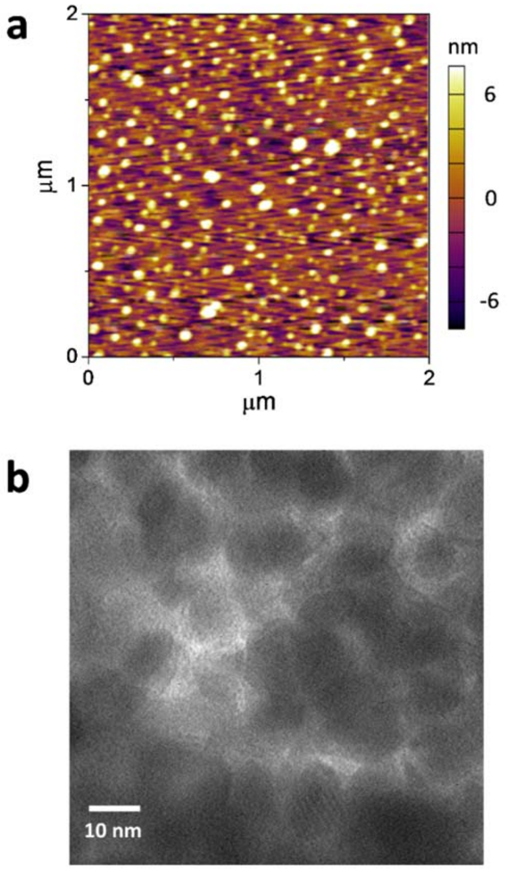

Supplementary Fig. 15 | (**a**) AFM image of un-treated medium $MAPbBr_3$ NCs film. (**b**) Representative TEM image of EDT-treated $MAPbBr_3$ NCs.

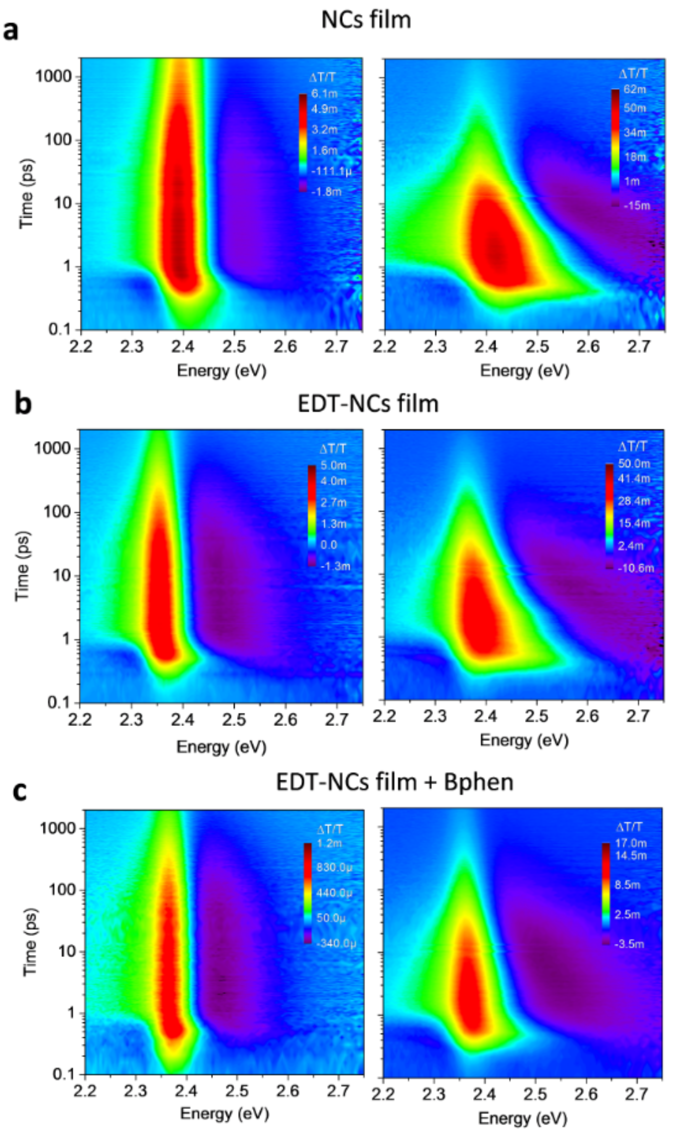

Supplementary Fig. 16 | Pseudocolor TA spectra for (**a**) medium $MAPbBr_3$ NCs film, (**b**) EDT-treated NCs film and (**c**) on EDT-treated NCs film/Bphen bilayer at low pump fluence (left panel) with initially generated $<N_0>$ ~0.1 and high pump fluence (right panel) with $<N_0>$ ~2.5. It is obvious that the high energy tails are reduced for EDT-NCs/Bphen following 3.1 eV photoexcitation.

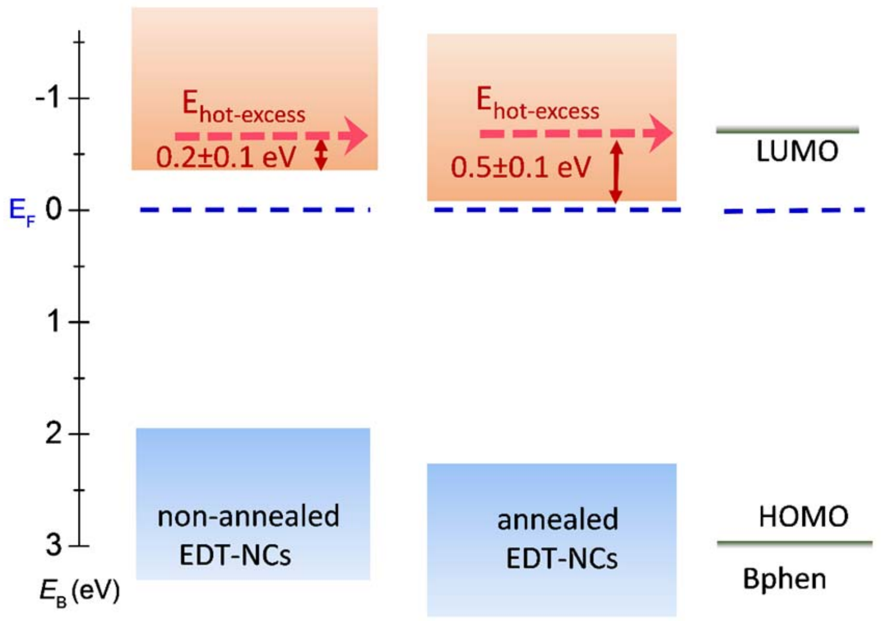

Supplementary Fig. 17 | Flat-band energy level alignment as determined from the UPS and UV-VIS spectroscopy measurements for non-annealed, annealed EDT-NCs films and Bphen - illustrated for the case of hot-electron extractions. Excess energy of extracted hot-electrons ($E_{hot-excess}$) was determined from the band offset between the conduction band minimum of NCs and the LUMO of Bphen.

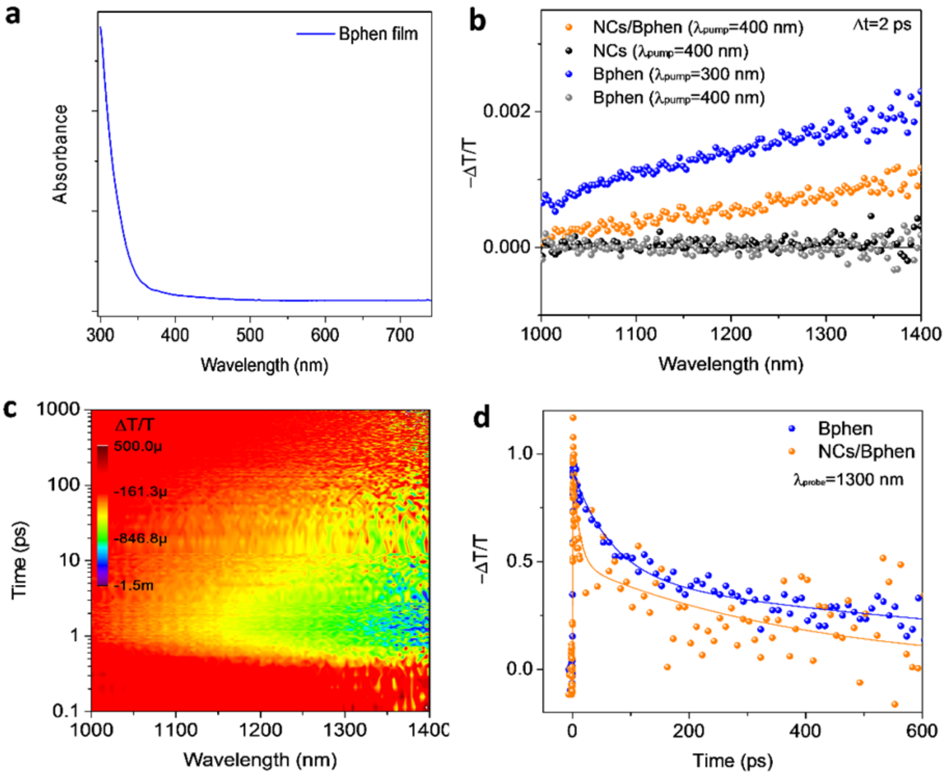

Supplementary Fig. 18 | (**a**) Linear absorption spectra of Bphen film on glass. (**b**) Negative TA spectra of Bphen (blue dot: 300 nm pump with intensity of 20 μJ cm^{-2}, gray dot: 400 nm pump with intensity of 40 μJ cm^{-2}), perovskites NCs (black dot, 400 nm pump with intensity of 15 μJ cm^{-2}) and EDT-NCs/Bphen (orange dot, 400 nm pump with intensity of 15 μJ cm^{-2}) at 2 ps after excitation. (**c**) Pseudocolor TA spectra of EDT-NCs/Bphen excited with 400 nm light at pump intensity of 15 μJ cm^{-2}. (**d**) Normalized negative TA spectra of Bphen excited with 300 nm light and EDT-NCs/Bphen pumped with 400 nm light and probed at 1300 nm. Solid lines are fitting curves with bi-exponential decay functions. See Supplementary Note 12 for discussions.

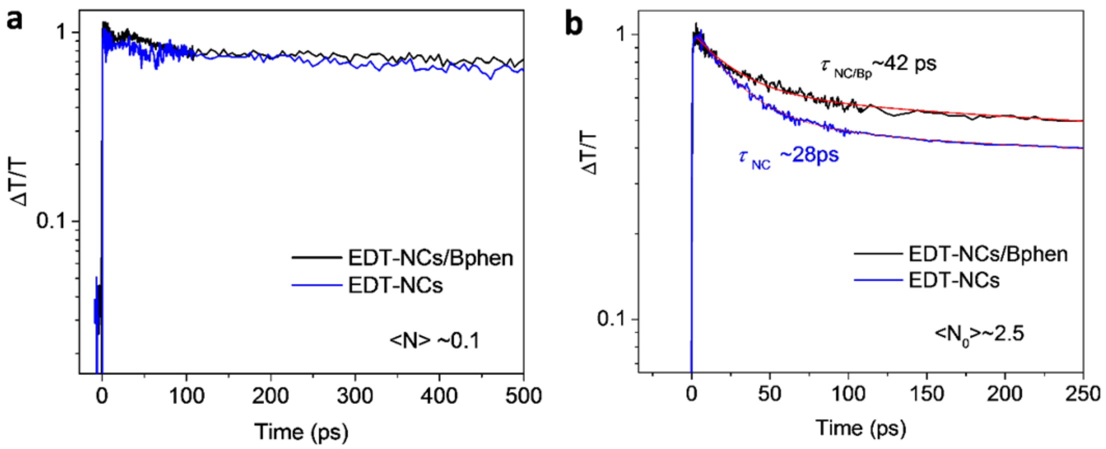

Supplementary Fig. 19 | Normalized band-edge bleaching dynamics of EDT-NCs film and EDT-NCs/Bphen bilayers under (**a**) low ($<N_0>$~0.1) and (**b**) high ($<N_0>$ ~2.5) pump fluence with 3.1 eV photoexcitation. See Supplementary Note 13 for discussions.

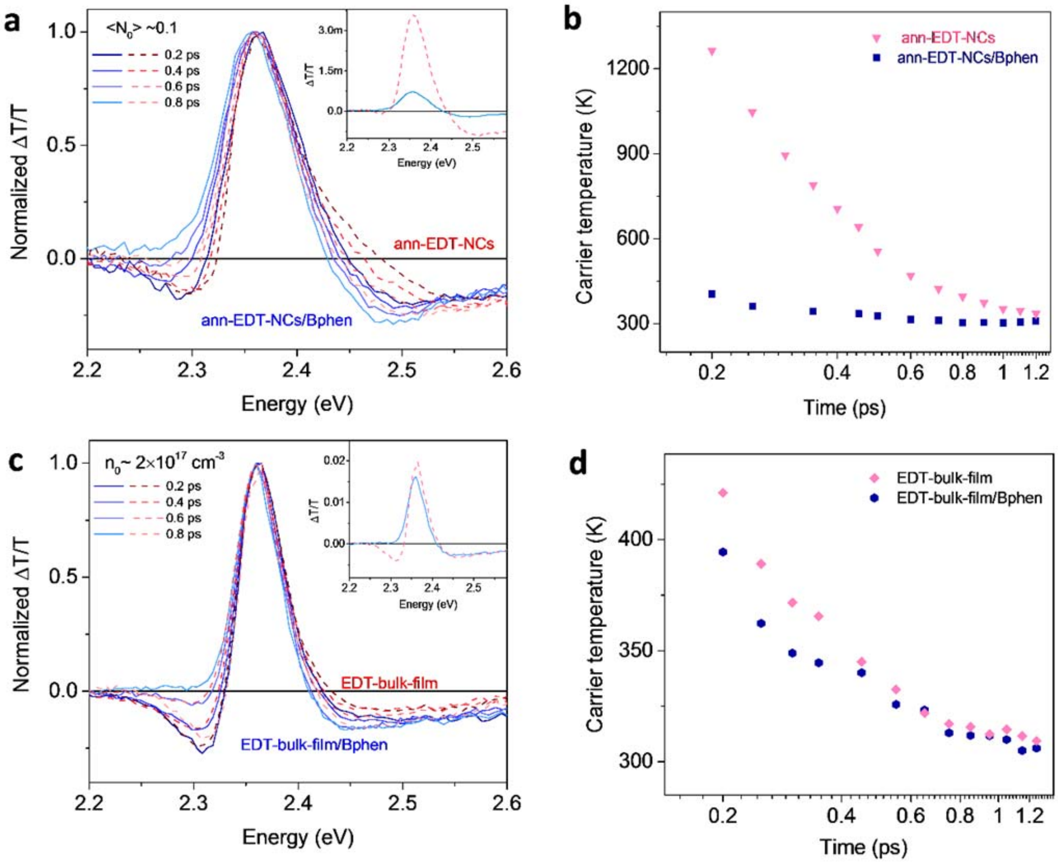

Supplementary Fig. 20 | (**a**) Normalized TA spectra for annealed EDT-treated medium $MAPbBr_3$ NCs film with (blue dashes) and without (red dashes) Bphen extraction layers at low fluence with $<N_0>$ ~ 0.1. Inset shows the un-normalized TA spectra at 0.8 ps. η_{hot} is determined to be ~83 %. (**b**) Extracted hot-carrier temperature as a function of delay time for two samples. (**c**) Normalized TA spectra for $MAPbBr_3$ bulk-film (~240 nm thick) with (blue dashes) and without (red dashes) Bphen extraction layers at low pump fluence with $2\times 10^{17} cm^{-3}$. Inset shows the un-normalized TA spectra at 0.8 ps. η_{hot} is determined to be ~16 %. (**d**) Extracted hot-carrier temperature as a function of delay time for two samples. Photoexcitation energy: 3.1 eV.

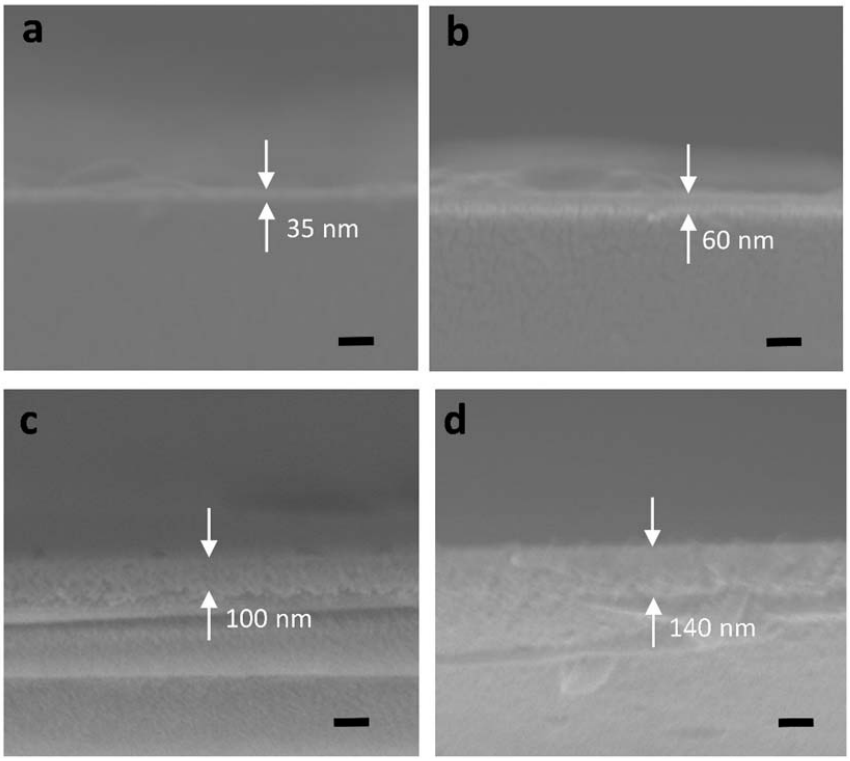

Supplementary Fig. 21 | (a)-(d) Cross-sectional SEM images of EDT-NCs film with different thickness. Scale bar is 100 nm.

Supplementary Table 1. List of the time delays after excitation for hot carriers cooled to 600 K# with the initially generated carrier densities and the excess energies above the band-edges in different materials. Slower delay times at equivalent photoexcitation excess energies and carrier densities are preferred.

materials	photoexcitation excess energy (eV)	low pump fluence		high pump fluence		Technique & Refs
		delay time after excitation (ps)	carrier density ($\times10^{17}cm^{-3}$)	delay time after excitation (ps)	carrier density ($\times10^{18}$ cm^{-3})	
CdSe nanorods (solution)	~ 1.1	~ 0.8	~55	~ 3	~22	TRPL[1]
$MAPbI_3$ thin films	~0.7	~ 0.5	~6.4	~ 1.1	~6.4	TA[2]
$MAPbI_3$ thin films	~0.8	~0.3	~5.2	-	-	TA[3]
	~1.4	~ 0.7	~5.2	~1	~1.5	TA[3]
				~20	~6.0	
GaAs thin film	~1.7	-	-	~2	~6.0	TA[3]
InN thin film	~0.3	-	-	~1.4	~6.0	TA[4]
$MAPbBr_3$ bulk-films	~0.7	< 0.1	~2.1	~0.8	~15	TA (our work)
$MAPbBr_3$ NCs (solution)		~ 0.7 (s)	~15	~10 (s)	~37.5	
		~ 0.6 (m)	~2.6	~18 (m)	~6.5	
		~ 0.5 (l)	~1.4	~32 (l)	~3.5	
$MAPbBr_3$ NCs (thin film)		~ 0.8 (s)	~15	~9 (s)	~37.5	
		~ 0.6 (m)	~2.6	~16 (m)	~6.5	
		~ 0.5 (l)	~1.4	~28 (l)	~3.5	

(s) = small; (m) = medium; (l) =large.

For sake of clear and easy comparison of hot-carrier cooling lifetimes reported for different materials in the literature, the hot-carrier cooling lifetime is defined as the time interval from pulse excitation till the cooling of hot-carriers to 600 K, which is useful for hot-carrier solar-cells. This temperature is used as the benchmark because previous theoretical calculations[11,12] have shown that for $T_c > 600$ K, there is still an appreciable hot-carrier conversion efficiency (*i.e.*, > 40%) over a wide range of absorber bandgaps.

Supplementary Notes

Supplementary Note 1. *Weak confinement in $MAPbBr_3$ NCs*

The exciton Bohr radius a_B of $MAPbBr_3$ was reported to be ~2 nm[5]. Given that the radius of our NCs (from ~2.5 to 5.6 nm, see size distribution histogram in Supplementary Fig. 2) is larger than a_B, our NCs are thus in the weak confinement regime. Hence, the small blue shift of emission (from 525 to 517 nm, Supplementary Fig. 4) of NCs with reducing NC size is due to the weak confinement effect. In the weak confinement, the first exciton resonance of NCs as a function of NCs radius a can be written as[6]:

$$E_{1s} = E_{g0} + \frac{\mu}{M}\left(\frac{\pi a_B}{a}\right)^2 E_b \qquad \text{(S1)}$$

where E_{g0} is the bandgap energy without quantum confinement while the second term represents the confinement energy, μ is the electron-hole reduced mass, $M = m_e^* + m_h^*$, E_b is the exciton binding energy. Using equation S1 above and the reported values of the effective masses m_e^*~0.29, m_h^*~0.31,[7] the absorption edges of our NCs can be reasonably well-fitted (Supplementary Fig. 4c) to yield a bandgap of E_{g0} ~2.38 eV and binding energy of E_b ~50 meV, which is close to the reported values for $MAPbBr_3$ NCs[8]. These fitting results further validate the weak confinement in our perovskites NCs.

Supplementary Note 2. *Analysis of TA spectra*

The analysis and interpretation of the TA spectra of halide perovskites are well-documented in the literature[2, 3, 9]. Our TA spectra of $MAPbBr_3$ perovskites (bulk and nanocrystals) are similar with that of previous studies. The positive TA peak (at ~2.3 eV as shown in Supplementary Fig. 6) arose from ground states bleaching (GSB) due to the state-filling of the carriers at the band edge. At the high energy side of the GSB peak, the first slope (*i.e.*, the steeper one closer to the GSB peak) is dependent on the ground state transitions which affect its width and shape; while the second gentler slope of the high energy tail (*e.g.*, start at ~2.5 eV in Supplementary Fig. 6) is resulted from the hot-carrier distribution. The negative part (photoinduced absorption) of this high energy side is caused by the photoinduced change of the imaginary part of the refractive index[2]; while the negative part of the low energy side of the GSB is attributed to bandgap renormalization[2].

Hot-carrier temperature T_c is extracted by fitting the high-energy tail of the TA spectra with a Maxwell-Boltzmann function. When the difference between the carrier's energy and the Fermi level is large as compared to $\kappa_B T$ (*i.e.*, $E - E_f > \kappa_B T$), the Fermi-Dirac distribution function can be approximately described with an exponential – *i.e.*, a Maxwell-Boltzmann distribution:

$$\frac{1}{1+\exp\frac{E-E_f}{\kappa_B T}} \approx exp\frac{E_f - E}{\kappa_B T} \qquad \text{(S2)}$$

Approximating the Fermi–Dirac distribution by a Maxwell–Boltzmann distribution for hot-carrier energies $\gg E_f$ is a valid and generally accepted practice to extract the hot-carrier temperature T_c. For our intrinsic (untreated) perovskites NCs and bulk-film, the Fermi level is located between the valence and conduction band edge (~0.4 eV below the conduction band minimum from our UPS data (Fig. 4a)). Thus, the generated hot-carriers is far from the Fermi energy (~ 1 eV above). Although the Fermi level may shift slightly towards the band edge (~0.1 eV) under intense photoexcitation, the energy difference is still very large (*i.e.*, $E - E_f \gg \kappa_B T$ (~25 meV at room temperature)). Thus, the high energy tail of the TA spectrum can be fitted using a Maxwell-Boltzmann distribution to extract T_c.

Supplementary Note 3. *Determination of absorption cross-sections of NCs*
Based on the Poisson distribution of initial photon occupancies in NCs, the probability of a NC to contain i e-h pairs is given by $P_i = \frac{<N_0>^i}{i!} e^{-<N_0>}$,[10] where $<N_0>=J\sigma$ is the initially generated average number of e-h pairs per NC (J is the pump fluence, σ is the absorption cross-section of NC). When the delay time after photoexcitation is much longer than multicarrier recombination (*e.g.*, Auger recombination), the NCs will mainly recombine with single exciton emission, hence the late-time PL intensity is proportional to occupation probabilities of photoexcited NCs as $I_{PL} \propto (1 - P_0) = (1 - e^{-J\sigma})$. Supplementary Fig. 7 shows the pump fluence dependent TRPL of the pump fluence dependent TRPL was normalized at time t = 4 ns when multicarrier recombination is completed and PL intensity represents the emitting NCs with only one electron-hole pair. σ of NCs can be obtained by fitting the data with equation of $1 - e^{-J\sigma}$ (solid lines). The fitted σ are $8.5\pm0.5\times10^{-15}$, $3.2\pm0.2\times10^{-14}$ and $6.8\pm0.3\times10^{-14}$ cm^2 from small to large NCs, respectively.

Supplementary Note 4. *Discussion on hot-carrier lifetimes*

It is important to note the complex interplay of the hot-carrier cooling times due to several factors:

(i) the *pump energy* (*i.e.*, carriers' excess energy – typically, higher the excess energies lead to longer hot carrier lifetimes);

(ii) the *initial hot-carrier densities* (*i.e.*, typically higher carrier densities lead to longer hot carrier lifetimes); and

(iii) the *energy loss rate at a specific hot-carrier temperature* (as shown in Fig 2a in manuscript where the energy loss rate changes over several orders of magnitude for hot-carrier temperatures spanning from 1600 to 300K) – typically, lower hot carrier temperatures yield smaller energy loss rates. (It should be noted that the listed lifetimes below are the time intervals from pulse excitation until the cooling of hot-carriers reach **600 K.**)

Without specifying the above parameters/conditions, it is ***difficult to generalize and very unfair to compare*** the hot-carrier lifetimes among different materials. Furthermore, the measured hot-carrier lifetime could be limited by the time-resolution of the experimental techniques used, thereby yielding artificially longer lifetimes that are limited by the system temporal response rather than its intrinsic hot-carrier lifetime. For example, measurement of the hot-carrier lifetime by the TRPL technique using a streak camera or TCSPC system will be constrained by the system resolution of these equipments (*i.e.*, ~10 ps for most streak cameras, as high as ~1 ps for Hamamatsu systems and typically ~50 ps for TCSPC systems). On the other hand, the TA or fluorescence upconversion PL techniques have much higher system temporal response of <150 fs, which would identify more authentic hot carrier lifetimes of the material. Hence, due care must be taken for a fair comparison of the reported values in the literature.

To ensure a fair comparison of the hot-carrier temperature and cooling dynamics, we present an extensive compilation of the materials (to the best of our abilities) ***complete with*** the consideration of the abovementioned parameters (*i.e.*, carrier densities, carrier temperatures, pump energies and techniques) in Supplementary Table 1. Furthermore, it should be noted that the hot-carrier cooling lifetime in Table S1 is defined as the time interval from pulse excitation untill the cooling of hot-carriers reach **600 K** (for point (iii) above). This temperature is used as the benchmark because previous theoretical calculations[11, 12] have shown that for T_c > 600 K, there is still an appreciable hot-carrier conversion efficiency (*i.e.*, > 40%) over a wide range of absorber bandgaps). We also wish to highlight that as the hot carrier distribution approaches thermal equilibrium with the lattice (300 K), the energy-loss rate will become much slower (see Fig. 2a). Although these pseudo "hot-carriers" give rise to a long lifetime, they will in fact have little contribution to the operation of a hot-carrier solar-cell. Therefore, this should not be compared here.

Supplementary Note 5. *LO-phonon model*

The energy loss rates per carrier J_r was determined by extracted T_c with $-1.5k_b dT_c/dt$. J_r can be fitted with the following model:

$$J_r = \frac{3}{2}\frac{\hbar\varpi_0}{\tau_{LO}}\left(e^{\frac{\hbar\varpi_0}{\kappa T_a}} - e^{\frac{\hbar\varpi_0}{\kappa T_c}}\right)\frac{N_{LO}(T_a)}{N_{LO}(T_c)}\left(\frac{\kappa T_c}{\hbar\varpi_0}\right)^2 e^{-\frac{\hbar\varpi_0}{\kappa T_c}} \quad \text{(S3)}$$

where τ_{LO} is the characteristic LO-phonon decay time, T_a is the acoustic phonon temperature, $\hbar\varpi_0$ is the phonon energy (~42 meV) and $N_{LO}(T)$ is the LO-phonon occupation number at temperature T. The fitting from Fig. 2a yielded a comparable T_a for the $MAPbBr_3$ NCs (~310 K) and bulk-film (~305 K), while τ_{LO} is ~340 fs, 220 fs and 180 fs for small, medium and large NCs, respectively, in contrast to a fast τ_{LO} of ~ 150 fs for the bulk-film.

Supplementary Note 6. *Photo-bleaching build-up dynamics at band-edges*

Apart from the approach of fitting the high energy tail of PB peak to elucidate the hot-carrier cooling properties, an alternative method is to probe the intraband relaxation of the photoexcited carriers high above the band-edge. This can be achieved through monitoring the buildup of the band-edge bleach as the recombination of the band-edge carriers (~ns) is much slower than its intraband relaxation process (from several to tens of ps). This latter approach is commonly used for investigating the hot-carrier dynamics in strongly confined quantum colloidal semiconductor NCs[13-15] given the overlapping PB bands from the discrete energy levels make resolving their hot-carrier distribution extremely challenging. We apply this latter approach for a fair comparison of the hot-carrier cooling of perovskites NCs with that of conventional inorganic semiconductor NCs (*e.g.*, CdSe NCs).

Supplementary Note 7. *Auger-heating model*

Auger decay lifetimes of $MAPbBr_3$ NCs are extracted from the pump fluence dependent band-edge photobleaching dynamics (Supplementary Fig. 9f), which exhibit a sublinear dependence on the NC volume (V_{NC}) as $\tau_{Aug} \sim \sqrt{V_{NC}}$ (Fig. 3b). This behavior agrees with recent observations of biexciton Auger recombination in weakly confined $CsPbBr_3$ NCs[16], but contrasts with the linear dependence of τ_{Aug} on NC size for strongly confined systems[17]. The sublinear dependence can therefore be attributed to the weaker confinement in our perovskites NCs.

Given that Auger recombination is a three-particle process, the Auger-heating rate in NCs is therefore proportional to $\sim n^3$, where n is the effective carrier density at the band-edge. Hence, the evolution of the hot-carrier population can be described by the following equation:

$$\frac{dn_{hot}(t)}{dt} = -An_{hot} + Cn^3 \quad \text{(S4)}$$

where the first term represents the relaxation of the hot-carriers unrelated to Auger heating, and the second term corresponds to the Auger heating contribution; C refers to the Auger recombination coefficient of the carriers at the band-edge. Within the lifetimes of the hot-carriers, one can neglect single exciton recombination given its long lifetime (of several ns). As a first approximation, the band-edge carriers recombine through the dominant Auger process given by: $n(\mathrm{t}) \sim e^{-t/\tau_{Aug}}$. Direct integration of this equation yields the time evolution of:

$$n_{hot}(t) = n_{hot0}(1 - D)e^{-At} + De^{-3t/\tau_{Aug}} \quad \text{(S5)}$$

where n_{hot0} is the initial population of the generated hot-carriers and D equals to $C/(A - 3/\tau_{Aug})$. Equation S4 therefore predicts that the hot-carrier population decays bi-exponentially, with one of its lifetime corresponding to $\tau_{Aug}/3$.

Considering the Fermi-Dirac distribution of hot-carriers, the effective hot-carrier density can be calculated using the relation[3]:

$$n_{\mathrm{hot}} \sim \left(\frac{2m_e \kappa T_{\mathrm{c}}}{\pi \hbar^2}\right)^{\frac{3}{2}} e^{(E_{\mathrm{f}}-E_{\mathrm{c}})/\kappa T_{\mathrm{c}}} \quad \text{(S4)}$$

Fig. 3c shows the normalized calculated hot-carrier densities as a function of decay time at different pump fluences.

Supplementary Note 8. *Estimation of hot-carrier diffusion length*

The hot-carrier diffusion length in $MAPbBr_3$ can be estimated as follows. Firstly, the carrier's diffusion coefficient depends on the defect density of the fabricated material. The reported electron diffusion coefficient D ranges from ~1 cm^2s^{-1} for polycrystalline perovskite thin films[18] to 5 - 8 cm^2s^{-1} for bulk $MAPbBr_3$ at room temperature (~300 K)[19, 20]. Secondly, D also depends on the carrier temperatures[21] (T_c) in the relation $D = \mu\kappa_{\mathrm{B}}T_{\mathrm{c}}/e$. For NCs-film, taking 800 K as the average hot-carrier temperature, the lower D value of 1 cm^2s^{-1}, and hot-carrier lifetime of 1 ps at low pump fluence, the hot-carrier diffusion length obtained by $L = \sqrt{D_{\mathrm{hot}}\tau_{\mathrm{hot}}} \approx 16\,\mathrm{nm}$. The high pump fluence at hot-carrier lifetime of ~32 ps yields a diffusion length of $L \approx 90\,\mathrm{nm}$. Considering that our perovskites/Bphen sample was excited at the Bphen side, and the initial exponential carrier distribution in semiconductors after fs laser pulse excitation[22], higher concentration of hot-carriers in perovskites closest to the Bphen can thus be more easily injected into Bphen. For NCs-film, given that some Bphen molecules could penetrate into the upper layer of the NCs film, and the hot-carriers undergoing rapid hopping[23], the extracted ~70% hot-carrier transfer efficiency for ~35 nm thick NCs-film at low pump fluence is therefore reasonable. For bulk-film, taking 400 K as the average hot-carrier temperature, the higher D values of 5 cm^2s^{-1}, and hot-carrier lifetime of 0.15 ps at low pump fluence, the hot-carrier diffusion length will be $\approx 10\,\mathrm{nm}$. Therefore, the ~15% transfer efficiency for bulk-film is also reasonable."

Supplementary Note 9. *FTIR and XPS analysis on ligand exchange*

FTIR spectroscopy of EDT-treated $MAPbBr_3$ NPs revealed highly efficient removal of the original oleic acid and oleylamine ligands (Supplementary Fig. 14). The removal of these ligands is clearly observed from the reduction of the CH_2 stretching at 2921 and 2841 cm^{-1}. The complete removal of C=O stretch at 1710 cm^{-1} along with the disappearance of wagging vibration of N–H at 800 cm^{-1} and C-O-H bond at 1384 cm^{-1} further supports the EDT ligand exchange of oleic acid and oleylamine ligands[24, 25].

XPS analysis of Sulphur in non-annealed and post-annealed EDT-treated $MAPbBr_3$ NCs reveals two sets of S 2p doublets, with the $2p^{3/2}$ peak position at binding energies ~162.5 eV and ~164.2 eV (~162.7 eV and ~164.3 eV for post-annealed) arising from bound thiolate and unbounded thiol of EDT to the surface of the NC respectively[26] (Supplementary Fig. 14). The ratio of bound-to-unbound thiol groups in the NC without

post-annealing is ~1.04, which increases to ~1.47 with post-annealing of the NC at 70 ^{o}C. Thus, post-annealing treatment further increases the electronic coupling of EDT-NCs with Bphen.

Supplementary Note 10. *Effects of photocharged NCs and trions in NCs films on hot-carriers*

Supplementary Fig. 9e shows the comparison of photobleaching dynamics at the band-edges between medium NCs in solution and spin-coated film. From the exponential fitting (solid curves), the lifetime changes from ~ 4.5 to ~3 ns at low pump fluence, the acceleration maybe due to the existence of the photocharged NCs. At high pump fluence, another fast decay with lifetime of ~ 290 ps except for the Auger recombination emerges in the spin-coated NCs films, which can be attributed to trions (photocharged excitons). However, they only induced the broadening in the lower energy side of GSB (see the appearance of a bleaching tail at lower energy side for NCs-films in Supplementary Fig. 16 as compared with Supplementary Fig. 5 for NCs in solution in the pseudo color TA spectra). The reduced energy of trions may be due to the exciton-exciton interactions[26]. It is obvious that the trions in the NCs-film would not affect the dynamics of hot-carriers located at the higher energy side of the GSB.

Supplementary Note 11. *Control experiments to validate the hot-carrier transfer*

The $MAPbBr_3$ NCs film demonstrate similar hot-carrier cooling dynamics with/without the EDT-treatment. Without the EDT-treatment, the NCs demonstrate similar hot-carrier cooling dynamics with/without the Bphen extraction layer. Furthermore, we did not find any obvious change in the hot-carrier properties in the NCs films that underwent the exact same processing in the thermal evaporator except no Bphen layer was actually deposited.

Supplementary Note 12. *PIA signal of transferred charge carriers in Bphen*

For the pristine Bphen film, upon excitation above band-gap with 300 nm light (refer to the absorption spectrum in Supplementary Fig. 18a), there is a very broad photoinduced absorption (PIA) in NIR range (Supplementary Fig. 18b – blue dots), typical of organic semiconductors (*e.g.*, P3HT, PCBM). Its intensity increases gradually with increasing probe wavelength. We tentatively assign the PIA bands primarily to the absorption by the photogenerated radical anions and/or excited singlet absorption in Bphen. Control experiments with 400 nm excitation (below bandgap) of Bphen (at both low and high pump fluence) yielded a null (PIA) signature (Supplementary Fig. 18b –gray dots), indicating the absence of photogenerated radical anions and/or excited singlet absorption in Bphen.

For the EDT-NCs/Bphen sample (with NCs-film thickness ~ 50 nm), the NCs are selectively excited with 400 nm light. At low pump fluence, there is no measureable TA signal. This could be due to the even weaker TA signal (of the indirectly generated radical anions) that is below the detection limit of our TA setup (at 10^{-4} ΔT/T). At higher pump fluence (*i.e.*, at ~15 μJ cm^{-2}, corresponding to <N>~2.5), we observed a weak PIA band similar to pristine Bphen (Supplementary Fig. 18b – orange dots and Supplementary Fig. 18c). However, any further increase of pump power would lead to the degradation of the perovskite.

The control experiments also show that there is no TA signal from the EDT-NCs/Bphen sample under 500 nm excitation (*i.e.*, with negligible excess energies for photoexcited carriers in NCs) and NCs film alone under 400 nm excitation (Supplementary Fig. 18b – black dots) up to high pump fluence. These experiments therefore indicate that the observation of PIA in EDT-NCs/Bphen hybrids should be caused by the hot-charge carriers injection into Bphen from the NCs. Nonetheless, there is also the possibility of an alternative explanation of excited singlet absorption induced by hot-state energy transfer from NCs to Bphen.

Furthermore, the relaxation of PIA possesses one fast decay lifetime (~70ps for pristine Bphen, and ~25ps for NCs/Bphen) and one slow decay lifetime (~1ns for pristine Bphen, and ~0.5 ns for NCs/Bphen) (Supplementary Fig. 18d). The fast decay could be due to the carrier trapping to defects for pristine Bphen and additional electron back-transfer to the NCs for NCs/Bphen. The slow decay could be the recombination of radical anions/excitons in the Bphen and with holes in NCs in the NCs/Bphen hybrids.

Supplementary Note 13. *Estimation of back-electron transfer time*

The back transfer of injected electrons from Bphen to NCs would induce an increase of the relaxation time of electrons in NCs in the TA signal. We thus estimate a back-electron transfer rate ($\frac{1}{\tau_{\mathrm{bk}}}$) from the change of the relaxation rate of NCs' bandedge bleaching between EDT-NCs film ($\frac{1}{\tau_{\mathrm{NC}}}$) and EDT-NC/Bphen bilayers ($\frac{1}{\tau_{\mathrm{NC/Bp}}}$) by $\frac{1}{\tau_{\mathrm{bk}}} = \frac{1}{\tau_{\mathrm{NC}}} - \frac{1}{\tau_{\mathrm{NC/Bp}}}$.

At low pump fluence (<N_0>~0.1), the invariance in the relaxation dynamics for the EDT-NCs film and the EDT-NC/Bphen bilayers (Supplementary Fig. 19a) indicates a very small back-electron transfer rate and a long back transfer time beyond our measurement time window, which may be due to the rapid trapping and localization that hinder the carriers from drifting back to the NCs film. The long τ_{bk} therefore is beneficial for the hot-electron injection. At high pump fluence (<N_0> ~2.5), there is an obvious lengthening of $\tau_{\mathrm{NC/Bp}}$ (Supplementary Fig. 19b). Using the above relation, the estimated back-electron time τ_{bk} is ~80 ps. The reduced back-electron transfer time (*i.e.*, increased back-

electron transfer rate) is consistent with the reduced hot-electron injection efficiency from 72% at $\langle N_0\rangle$~0.1 to 58% at $\langle N_0\rangle$ ~2.5.

Supplementary References

1. Achermann, M., Bartko, A.P., Hollingsworth, J.A. & Klimov, V.I. The effect of Auger heating on intraband carrier relaxation in semiconductor quantum rods. *Nat Phys* **2**, 557-561 (2006).
2. Price, M.B. et al. Hot-carrier cooling and photoinduced refractive index changes in organic-inorganic lead halide perovskites. *Nat Commun* **6**, 8420(2015).
3. Yang, Y. et al. Observation of a hot-phonon bottleneck in lead-iodide perovskites. *Nat Photonics* **10**, 53-59 (2016).
4. Wen, Y.C., Chen, C.Y., Shen, C.H., Gwo, S. & Sun, C.K. Ultrafast carrier thermalization in InN. *Appl Phys Lett* **89**, 232114-232114 (2006).
5. Tanaka, K. et al. Comparative study on the excitons in lead-halide-based perovskite-type crystals $CH_3NH_3PbBr_3$ $CH_3NH_3PbI_3$. *Solid State Commun* **127**, 619-623 (2003).
6. Dirote, E.V. Trends in nanotechnology research, Nova Publishers. (2004).
7. Melissen, S.T.A.G., Labat, F., Sautet, P. & Le Bahers, T. Electronic properties of $PbX_3CH_3NH_3$ (X = Cl, Br, I) compounds for photovoltaic and photocatalytic applications. *Phys Chem Chem Phys* **17**, 2199-2209 (2015).
8. Kumar, P., Muthu, C., Nair, V.C. & Narayan, K.S. Quantum Confinement Effects in Organic Lead Tribromide Perovskite Nanoparticles. *J Phys Chem C* **120**, 18333-18339 (2016).
9. Sum, T.C. et al. Spectral Features and Charge Dynamics of Lead Halide Perovskites: Origins and Interpretations. *Accounts Chem Res* **49**, 294-302 (2016).
10. Klimov, V.I. Optical nonlinearities and ultrafast carrier dynamics in semiconductor nanocrystals. *J Phys Chem B* **104**, 6112-6123 (2000).
11. Ross, R.T. & Nozik, A.J. Efficiency of Hot-Carrier Solar-Energy Converters. *J Appl Phys* **53**, 3813-3818 (1982).
12. Takeda, Y. et al. Hot carrier solar cells operating under practical conditions. *J Appl Phys* **105**, 074905(2009).
13. Klimov, V.I. & McBranch, D.W. Femtosecond 1P-to-1S electron relaxation in strongly confined semiconductor nanocrystals. *Phys Rev Lett* **80**, 4028-4031 (1998).
14. Yu, P.R., Nedeljkovic, J.M., Ahrenkiel, P.A., Ellingson, R.J. & Nozik, A.J. Size dependent femtosecond electron cooling dynamics in CdSe quantum rods. *Nano Lett* **4**, 1089-1092 (2004).
15. Yang, J., Hyun, B.R., Basile, A.J. & Wise, F.W. Exciton Relaxation in PbSe Nanorods. *Acs Nano* **6**, 8120-8127 (2012).
16. Nikolay S. Makarov, S.G., Oleksandr Isaienko, Wenyong Liu, István Robel, and Victor I. Klimov. Spectral and dynamical properties of single excitons, biexcitons, and trions in cesium-lead-halide perovskite quantum dots. *Nano Lett* **16**, 2349-2362 (2016).
17. Klimov, V.I., Mikhailovsky, A.A., McBranch, D.W., Leatherdale, C.A. & Bawendi, M.G. Quantization of multiparticle Auger rates in semiconductor quantum dots. *Science* **287**, 1011-1013 (2000).
18. Li, Y. et al. Direct Observation of Long Electron-Hole Diffusion Distance in $CH_3NH_3PbI_3$ Perovskite Thin Film. *Sci. Rep.* **5**, 14485(2015).
19. Wu, B. et al. Discerning the Surface and Bulk Recombination Kinetics of Organic-Inorganic Halide Perovskite Single Crystals. *Adv Energy Mater* **6**, 1600551(2016).
20. Yamada, T. et al. Fast Free-Carrier Diffusion in CH3NH3PbBr3 Single Crystals Revealed by Time-Resolved One- and Two-Photon Excitation Photoluminescence Spectroscopy. *Adv Electron Mater* **2**, 1500290 (2016).
21. Yoffa, E.J. Dynamics of Dense Laser-Induced Plasmas. *Phys Rev B* **21**, 2415-2425 (1980).

22. Hulin, D. et al. Energy-Transfer during Silicon Irradiation by Femtosecond Laser-Pulse. *Phys Rev Lett* **52**, 1998-2001 (1984).
23. Gao, Y. et al. Enhanced Hot-Carrier Cooling and Ultrafast Spectral Diffusion in Strongly Coupled PbSe Quantum-Dot Solids. *Nano Lett* **11**, 5471-5476 (2011).
24. Cooper, J.K., Franco, A.M., Gul, S., Corrado, C. & Zhang, J.Z. Characterization of Primary Amine Capped CdSe, ZnSe, and ZnS Quantum Dots by FT-IR: Determination of Surface Bonding Interaction and Identification of Selective Desorption. *Langmuir* **27**, 8486-8493 (2011).
25. Gao, Y.N. et al. Photoconductivity of PbSe Quantum-Dot Solids: Dependence on Ligand Anchor Group and Length. *Acs Nano* **6**, 9606-9614 (2012).
26. Cao, Y., Stavrinadis, A., Lasanta, T., So, D. & Konstantatos, G. The role of surface passivation for efficient and photostable PbS quantum dot solar cells. *Nature Energy* **1**, 16035 (2016).
